# Profiling of Key Hub Genes Using a Two-State Weighted Gene Co-Expression Network of ‘Jao Khao’ Rice under Soil Salinity Stress Based on Time-Series Transcriptome Data

**DOI:** 10.3390/ijms252011086

**Published:** 2024-10-16

**Authors:** Prasit Khunsanit, Kitiporn Plaimas, Supachitra Chadchawan, Teerapong Buaboocha

**Affiliations:** 1 Program in Biotechnology, Faculty of Science, Chulalongkorn University, Bangkok 10330, Thailand; khunsanit.prasit@gmail.com; 2 Center of Excellence in Molecular Crop, Department of Biochemistry, Faculty of Science, Chulalongkorn University, Bangkok 10330, Thailand; 3 Department of Mathematics and Computer Science, Faculty of Science, Chulalongkorn University, Bangkok 10330, Thailand; kitiporn.p@chula.ac.th; 4 Center of Excellence in Environment and Plant Physiology, Department of Botany, Faculty of Science, Chulalongkorn University, Bangkok 10330, Thailand; supachitra.c@chula.ac.th; 5 Omics Sciences and Bioinformatics Center, Faculty of Science, Chulalongkorn University, Bangkok 10330, Thailand

**Keywords:** network-based analysis, rice, RNA-sequencing, salt tolerance, transcriptomic, two-state co-expression network, weighted co-expression network

## Abstract

RNA-sequencing enables the comprehensive detection of gene expression levels at specific time points and facilitates the identification of stress-related genes through co-expression network analysis. Understanding the molecular mechanisms and identifying key genes associated with salt tolerance is crucial for developing rice varieties that can thrive in saline environments, particularly in regions affected by soil salinization. In this study, we conducted an RNA-sequencing-based time-course transcriptome analysis of ‘Jao Khao’, a salt-tolerant Thai rice variety, grown under normal or saline (160 mM NaCl) soil conditions. Leaf samples were collected at 0, 3, 6, 12, 24, and 48 h. In total, 36 RNA libraries were sequenced. ‘Jao Khao’ was found to be highly salt-tolerant, as indicated by the non-significant differences in relative water content, cell membrane stability, leaf greenness, and chlorophyll fluorescence over a 9-day period under saline conditions. Plant growth was slightly retarded during days 3–6 but recovered by day 9. Based on time-series transcriptome data, we conducted differential gene expression and weighted gene co-expression network analyses. Through centrality change from normal to salinity network, 111 key hub genes were identified among 1,950 highly variable genes. Enriched genes were involved in ATP-driven transport, light reactions and response to light, ATP synthesis and carbon fixation, disease resistance and proteinase inhibitor activity. These genes were upregulated early during salt stress and RT-qPCR showed that ‘Jao Khao’ exhibited an early upregulation trend of two important genes in energy metabolism: RuBisCo (*LOC_Os10g21268*) and ATP synthase (*LOC_Os10g21264*). Our findings highlight the importance of managing energy requirements in the initial phase of the plant salt-stress response. Therefore, manipulation of the energy metabolism should be the focus in plant resistance breeding and the genes identified in this work can serve as potentially effective candidates.

## 1. Introduction

High soil salinity significantly affects rice cultivation by impeding root water absorption, leading to osmotic stress in plant cells. Moreover, an excessive influx of NaCl can exacerbate ionic and oxidative stress [1]. Understanding the molecular mechanisms underlying salt tolerance is essential for enhancing the resilience of rice varieties to salinity, particularly in areas affected by soil salinization. Salt tolerance is governed by numerous genes or quantitative trait loci (QTLs), which collaborate via co-regulation and/or co-expression to alleviate salinity-induced stress via intricate molecular mechanisms.

Transcript–transcript association network analysis plays a crucial role in the elucidation of the molecular mechanisms of a biological system in systems biology studies [2]. With the progression of deep genome sequencing, RNA-sequencing has been used to investigate gene expression in rice under particular abiotic stimuli, such as salinity [3]. Weighted gene co-expression network analysis (WGCNA), a molecular biological correlation network analysis method, uses transcriptome data to analyze intricate gene-regulatory networks by grouping co-expressed genes into adjacency modules and classifying them based on functional and pathway enrichment. This approach relies on a strong pairwise correlation of transcript levels between specific time points. WGCNA was initially developed by Langfelder and Horvath [4]. WGCNA has proven to be effective in identifying hub genes associated with both biotic and abiotic stresses. For example, in Arabidopsis, network centrality analyses for identifying key transcription factors crucial in drought stress conditions revealed 117 transcription factors with hub-like properties [5]. Similarly, in rice seedlings, 17 potential candidates associated with drought, salinity, and heat stress were identified by analyzing centrality properties and densely interconnected interactions [6].

Two-state GCNA was adopted to identify key genes in the global (encompassing transcriptome data from two conditions), the normal-state (containing transcriptome data from seedlings grown under normal conditions), and the stress-state (containing transcriptome data from stress conditions) networks. The global network served as an overarching network. The two-state networks facilitated analysis of the centrality changes from the normal to the saline conditions. Using two-state WGCNA, Sonsungsan, et al. [7] identified key salt tolerance genes in ‘Luang Pratahn’, a Thai rice variety, and reported that more than 44% of the key genes were related to salt stress and various stress responses. Chutimanukul, et al. [8] performed WGCNA to detect potential salt-tolerance genes in chromosome segment substitution lines of ‘Khao Dawk Mali 105’. *OsBTBZ1* (LOC_Os01g66890), initially identified as a potential salt-tolerance gene, was further characterized and found to function as a salt-tolerance gene operating via abscisic acid (ABA)-dependent pathways [9].

Since salt tolerance is a multigene trait involving several cellular processes and dependent on the plant genetic background, comprehensive identification of key genes involved and their variations among different varieties will lead to complete understandings on the mechanisms of salinity tolerance. The Thai rice variety ‘Jao Khao’ is salt-tolerant during both the seedling and flowering stages and maintains high yields under saline conditions [10,11]. In the present study, we conducted two-state WGCNA of ‘Jao Khao’ rice at the seedling stage using transcriptomic time-series data to compare responses under normal and saline conditions. The aim of this study was to identify major cellular processes and crucial hub genes implicated in salt stress.

## 2. Results

### 2.1. Salt-Stress Related Phenotypes of ‘Jao Khao’ under Saline Soil Conditions

**Figure 1:**
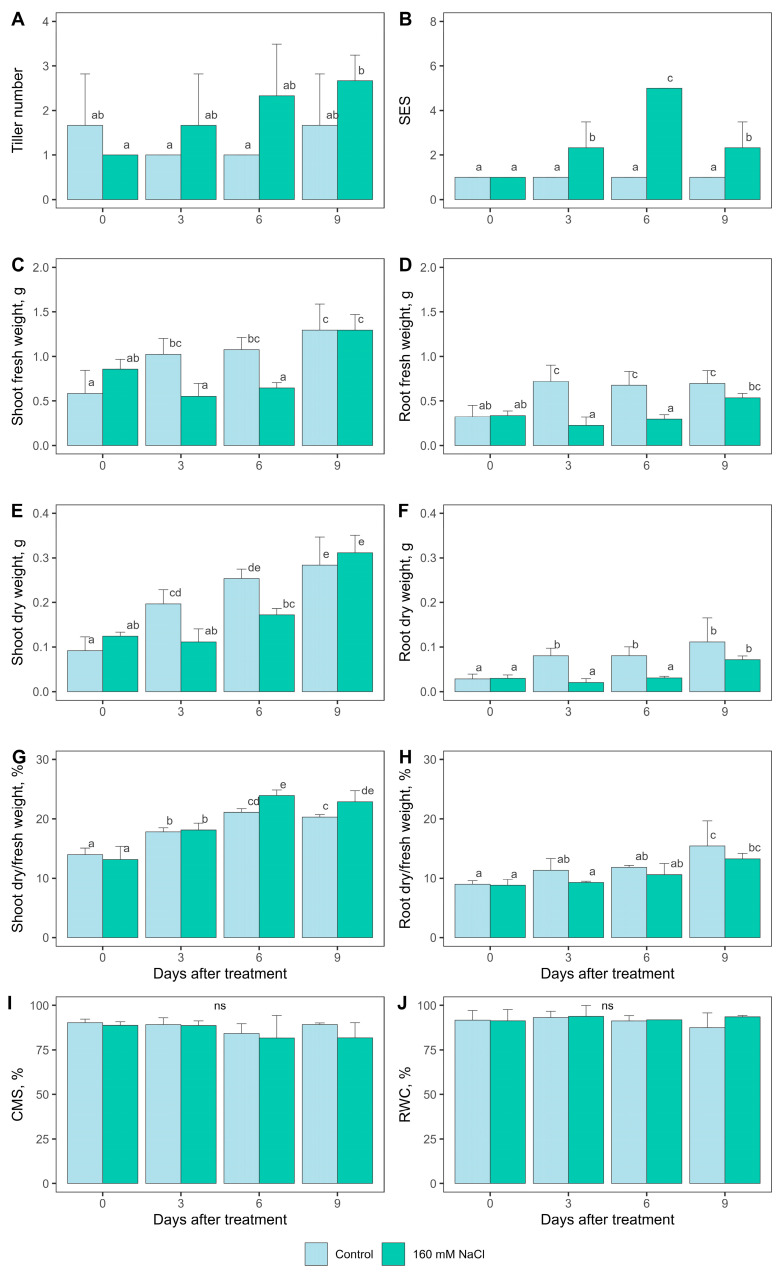
Comparison of phenotypic traits between normal and saline conditions of ‘Jao Khao’. (**A**) tiller number, (**B**) SES, (**C**) shoot fresh weight, (**D**) root fresh weight, (**E**) shoot dry weight, (**F**) root dry weight, (**G**) shoot dry-to-fresh weight ratio, (**H**) root dry-to-fresh weight ratio, (**I**) CMS, and (**J**) RWC. Data are presented as means ± SD (n = 3). Statistical significance was determined using the Duncan multiple range test. Significant differences (*p* ≤ 0.05) are indicated by different letters. ns: not significant.

We determined the tiller number, plant weight, salt injury evaluation system (SES), cell membrane stability (CMS), and relative water content (RWC) at days 0, 3, 6, and 9 under normal and saline (160 mM NaCl) soil conditions. All phenotypic data are presented in Figure 1 and Supplementary Table S1 and Figure S1. In response to salt stress, the tiller number tended to increase from day 0 to day 9, and the SES score significantly increased from day 0 to day 6 but decreased on day 9 (Figure 1A,B). Shoot and root fresh weights and dry weights were lower in the saline treatment group on days 3 and 6 but did not significantly differ on day 9 (Figure 1C–F). The percentages of dry/fresh weight, which represent the proportions of dry mass and water content, indicated that the shoots of plants under normal conditions contained slightly more water (lower mass percentage) than those of plants under saline conditions during days 6–9, whereas the roots did not significantly differ in terms of water content between the two conditions (Figure 1G,H). Unlike ‘Pokkali’ and ‘IR29’ (Supplementary Figure S2), the CMS and RWC of ‘Jao Khao’ did not change under saline conditions during the 9-day treatment (Figure 1I,J).

### 2.2. Leaf Greenness and Chlorophyll Fluorescence of ‘Jao Khao’ under Saline Soil Conditions

**Figure 2:**
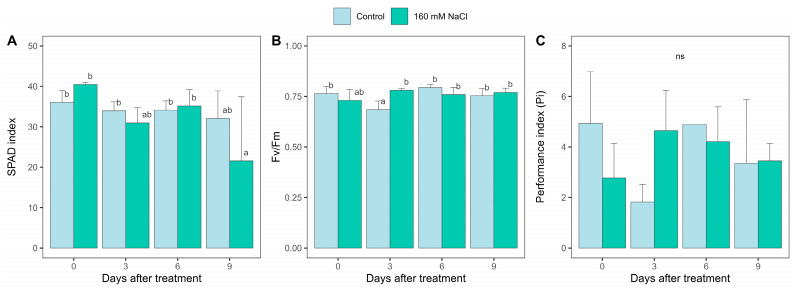
Leaf greenness and chlorophyll fluorescence of ‘Jao Khao’ compared between that of control and salt conditions. (**A**) SPAD index, (**B**) Maximum PSII efficiency (Fv/Fm), and (**C**) Performance index (Pi). Data are presented as means ± SD (n = 3). Statistical significance was determined using the Duncan multiple range test. Significant differences (*p* ≤ 0.05) are indicated by different letters. ns: not significant.

According to the SPAD index, which represents leaf greenness, ‘Jao Khao’ can maintain the leaf greenness during the 9-day salt stress (Figure 2A), while the indices of ‘IR29’ and ‘Pokkali’ markedly decreased in days 6 and 9, respectively, under salt stress (Supplementary Figure S3A,B). Similarly, ‘Jao Khao’ seedlings were able to maintain a high maximum quantum yield of PSII (Fv/Fm), compared to that of normal conditions throughout the 9-day period of salt stress (Figure 2B), and significantly higher than those of ‘IR29’ and ‘Pokkali’, which markedly decreased on days 6 and 9, respectively (Supplementary Figure S3C,D). Consistently, the Performance index (Pi), which indicates alterations in the photosynthetic apparatus of plants under stress, based on light energy absorption [8,12,13] of ‘Jao Khao’ was maintained until day 9 (Figure 2C), which was also higher than those of ‘IR29’ and ‘Pokkali’ (Supplementary Figure S3E,F).

### 2.3. Overview of Two-State Network Analysis

**Figure 3:**
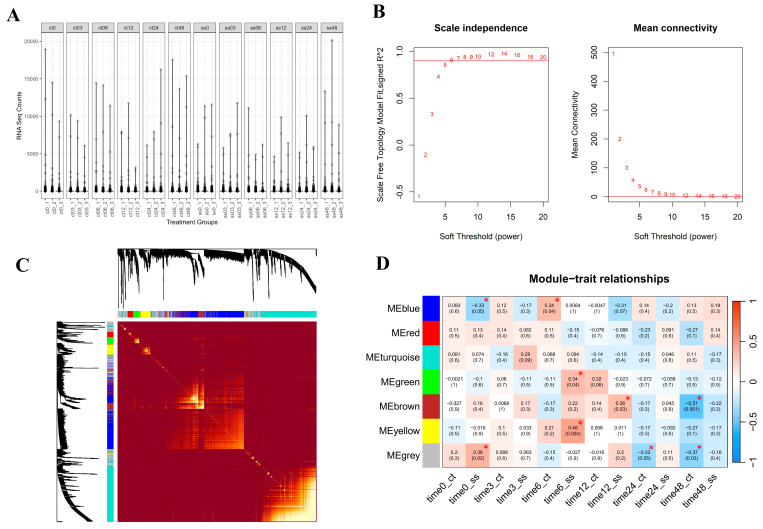
Normalized read counts of 36 RNA-sequencing library (**A**), scale-free topology and mean connectivity (the horizontal red line was at *R*^2^ = 0.9) (**B**), the heatmap of topological overlapping matrix (TOM) plot visualizing the strength of the connections (similarity) between genes with the bright yellow color indicating genes with more connections or shared neighbors in the network and the colors representing modules displayed on both axes (**C**), and module–trait relationships (MTR) (**D**). The colors of modules include blue, red, turquoise, green, brown, yellow and grey. The letters ct and ss indicate control conditions and salt stress conditions, respectively. ME: Module Eigengene, a representative of gene expression levels in a cluster of co-expressed genes. Significant differences (*p* ≤ 0.05) are indicated by *.

We constructed three networks: a global network encompassing all data, covering both the normal and salinity states, and separate networks for the normal and saline states. First, all genes were normalized and qualified, after which WGCNA was used to create the networks. The global network served as an overview of the network analysis, including module detection. For the normal- and saline-state networks, genes were not clustered into modules; rather, node centralities (DG, BW, CN, and CC) were calculated. To identify key genes in each module, changes in the centrality values from the normal- to the saline-state network were assessed. In total, 27,106,303 read counts and 38,993 genes were obtained from the 36 libraries. The raw read counts (Figure 3A) were normalized using DESeq2, (version 1.44.0) and after filtering out genes with low variation across all conditions, 1950 genes that passed the 95th percentile threshold were retained. In WGCNA, the soft threshold was determined based on a power of 10 (Figure 3B) selected for calculating the adjacency matrix. The heatmap of topological overlapping matrix plot (Figure 3C) shows the clustering of genes with similar expression patterns into modules. In this context, modules and clusters represent the same concept, that is, genes grouped together based on shared expression profiles. The minimum number of genes per module was set to 35, resulting in seven modules: grey (362 genes), green (59 genes), turquoise (610 genes), yellow (113 genes), red (48 genes), blue (567 genes), and brown (191 genes) (Table 1 and Figure 3C).

### 2.4. Determination of Node Centralities

The network properties of the three networks were calculated based on the WGCNA results and a summary of the network statistics is provided in Table 2. To include all connections, edge weights were set to 0.1 or higher [7]. The saline-state network had a higher number of nodes (1351 nodes) than the normal-state network (1204 nodes), whereas the global network had an even lower number of nodes (909 nodes). The normal-state network had 59,749 edges, an average degree of 206.420, and 52 connections per node. In contrast, the salinity-state network had 54,090 edges, a lower average degree of 120.038, and only 21 connections per node. The diameter was the largest in the saline-state network (16) and the smallest in the global network (7). These findings suggested that during salt stress, ‘Jao Khao’ rice exhibited altered expression of genes (nodes), but the genes were less connected as indicated by the decreased number of edges, which led to an increased network diameter. A smaller diameter typically indicates a more compact and closely connected network, whereas a larger diameter suggests a more dispersed or loosely connected network.

**Figure 4:**
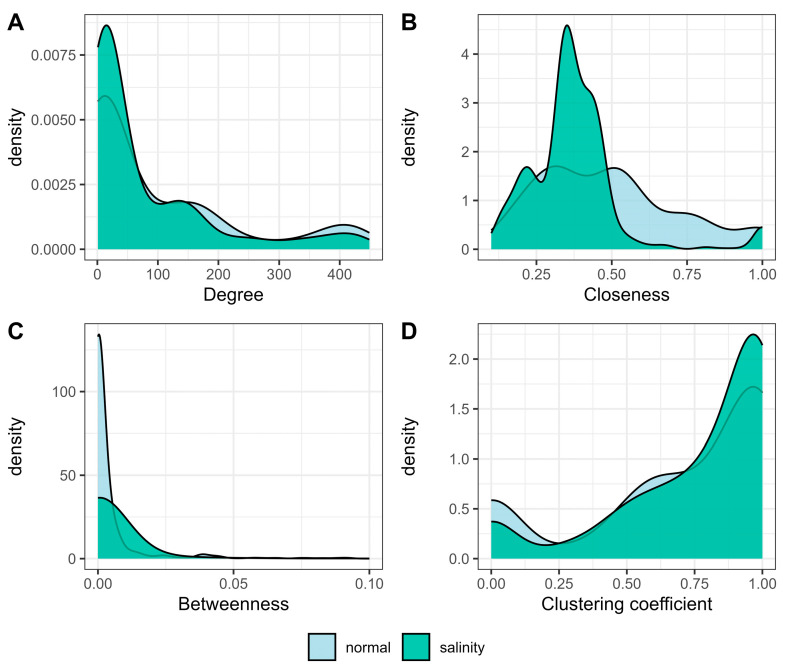
Comparison of the distribution of centrality between the normal-state and saline-state networks for (**A**) degree, (**B**) closeness, (**C**) betweenness, and (**D**) the clustering coefficient.

A comparison of the centrality distribution between the normal- and saline-state networks is shown in Figure 4. The density of degree values increased during salt exposure in the range of 0–70 (genes with low connections) but decreased in the range of 200–400 (genes with high connections) (Figure 4A). Similarly, the density of closeness increased in the range of 0–0.5 but decreased in the range of 0.5–1 (Figure 4B). The densities of betweenness clearly decreased under salt stress (Figure 4C) and clustering coefficients exhibited left-skewed distribution in both networks (Figure 4D).

### 2.5. Module Relationships with Multiple Time Points

The module–trait relationships (MTRs) were used to explain the correlation between genes in each module and time points. MTRs were established for the global network, as depicted in Figure 3D. ‘Jao Khao’ rice, which is highly resistant to salinity and, under saline conditions, has a similar phenotype to that under normal growth conditions (Figure 1), exhibited a few significant correlations between module genes and time points. However, we observed a significant upregulation of genes in three modules related to saline treatment at specific time points. Genes in the yellow module were upregulated at 6 h (MTRs = 0.46, *p* = 0.004), as were those in the green module (MTRs = 0.34, *p* = 0.04), whereas those in the brown module were upregulated at 12 h (MTRs = 0.36, *p* = 0.03). These results suggest that the expression of genes in these modules was upregulated early. Details of the relationship between genes in this module and salt stress are provided in Section 3. This observation correlates with the summarized network properties presented in Table 2, which indicate that salt stress in ‘Jao Khao’ rice initiates an alteration in gene expression as suggested by the increase in the number of nodes, but with fewer connections among them.

### 2.6. Functional and Pathway Enrichment Analyses

**Figure 5:**
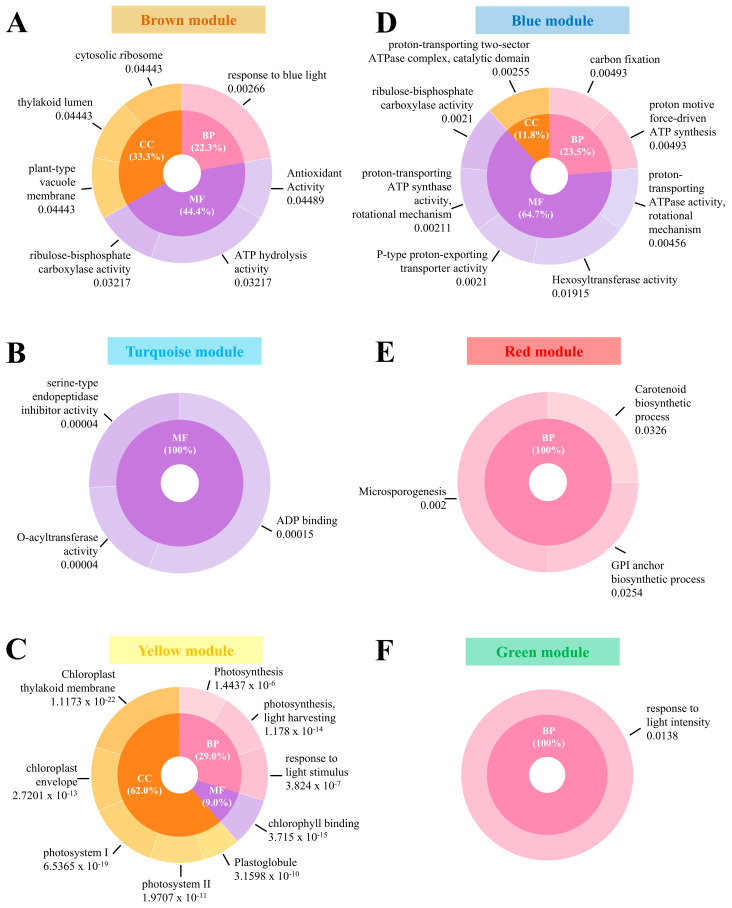
Gene Ontology (GO) enrichment analysis results for each module. *p*-values were adjusted using the Benjamini–Hochberg correction. (**A**) Brown module, (**B**) turquoise module, (**C**) yellow module, (**D**) blue module, (**E**) red module, and (**F**) green module. GO terms include biological process (BP), cellular component (CC), and molecular function (MF).

To elucidate the biological relevance of the gene expression data, gene enrichment analysis was conducted using genes from each module by mapping them to the rice annotation database using the clusterProfiler function in R (version 4.4.1) [14]. GO terms were used to categorize the genes into BP, CC, and MF categories. GO terms were identified for genes within all modules except the grey module (Figure 5 and Supplementary Table S3). The green module was largely associated with BP (100%), including response to light intensity. The turquoise module was mainly involved in MF (100%), including serine-type endopeptidase inhibitor activity, *O*-acyltransferase activity, and ADP-binding. The yellow module was correlated with CC (62%), including photosystems and chloroplast thylakoid membrane; BP (29%), including photosynthesis and response to light stimulus; and MF (9%), i.e., chlorophyll binding. The red module was exclusively associated with BP (100%), including microsporogenesis, carotenoid biosynthetic process, and glycosyl-phosphatidylinositol (GPI) anchor biosynthetic process. The blue module was associated with MF (65%), including hexosyltransferase activity, RuBisCo activity, and ATP synthase activity; BP (24%), including ATP synthesis and carbon fixation; and CC (12%), including ATPase complex. Finally, the brown module was related to MF (44%), including RuBisCo activity, ATP hydrolysis activity, and antioxidant activity; CC (33%), including thylakoid lumen, vacuole membrane, and ribosome; and BP (22%), including the response to blue light.

**Figure 6:**
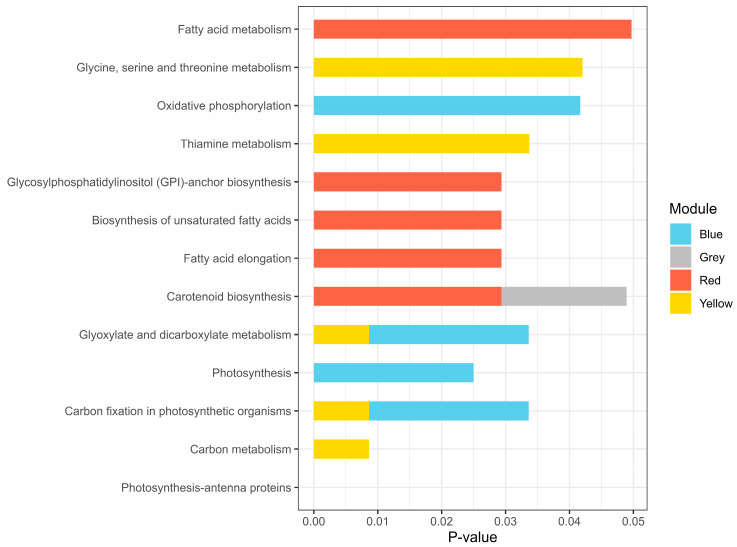
Kyoto Encyclopedia of Genes and Genomes (KEGG) pathway enrichment results for each module. *p*-values were adjusted using the Benjamini–Hochberg correction.

The results of KEGG pathway enrichment analysis of the genes each module are provided in Figure 6 and Supplementary Table S4. The yellow and blue modules exhibited related pathways including glyoxylate and dicarboxylate metabolism, carbon fixation in photosynthetic organisms, oxidative phosphorylation, photosynthesis, and carbon metabolism with some of them overlapped. The yellow module was also enriched in glycine, serine and threonine metabolism, and thiamine metabolism. The red and grey modules shared the carotenoid biosynthesis pathway. Additionally, the red module exhibited several terms related to fatty acids. These findings suggest that the significant genes within these modules are primarily involved in photosynthesis-related pathways and carbon metabolism.

### 2.7. Investigation of Modular Hub Genes

**Figure 7:**
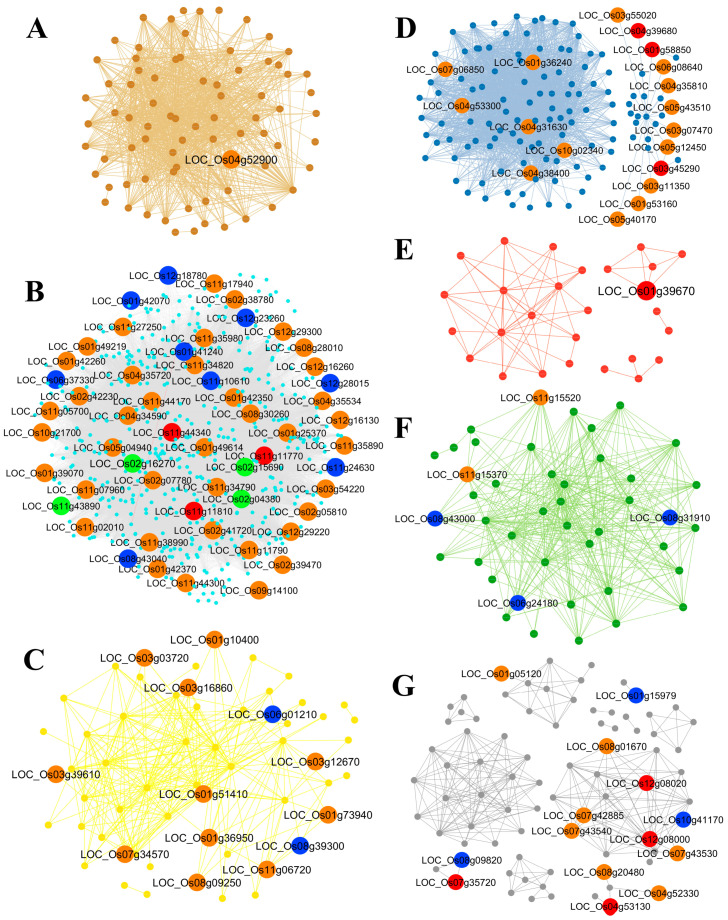
Gene networks and key genes were identified based on the centrality change between the two states (normal and saline) and mapped to the global state network. (**A**) Brown module, (**B**) turquoise module, (**C**) yellow module, (**D**) blue module, (**E**) red module, (**F**) green module, and (**G**) grey module. Small nodes and edges are colored according to the module they belong to. Large nodes represent key genes detected based on DG, BW, CN, and CC centrality, with combinations of 1, 2, 3, and 4 centrality measures, which are indicated in bright colors: green, blue, orange, and red, respectively.

Analysis focusing on the differences in node centrality between the normal- and saline-state networks identified 111 key genes, which were mapped onto the global network. The genes were distributed across the grey (14 genes), green (5 genes), turquoise (59 genes), yellow (13 genes), red (1 gene), blue (18 genes), and brown (1 gene) modules as indicated in Table 2 and Supplementary Table S2, and across the 12 chromosomes of the rice genome as illustrated in Supplementary Figure S4. Notably, key hub genes were found on all chromosomes. Genes within the same module were often located in close proximity on a chromosome. Chromosomes 11, 1, and 4 contained the highest numbers of key genes (23, 19, and 11 genes, respectively), whereas chromosome 5 had only one key gene. Key genes were mapped to the global state network as shown in Figure 7.

### 2.8. ‘Jao Khao’ Exhibits Early Upregulation of Genes Involved in Energy Metabolism

**Figure 8:**
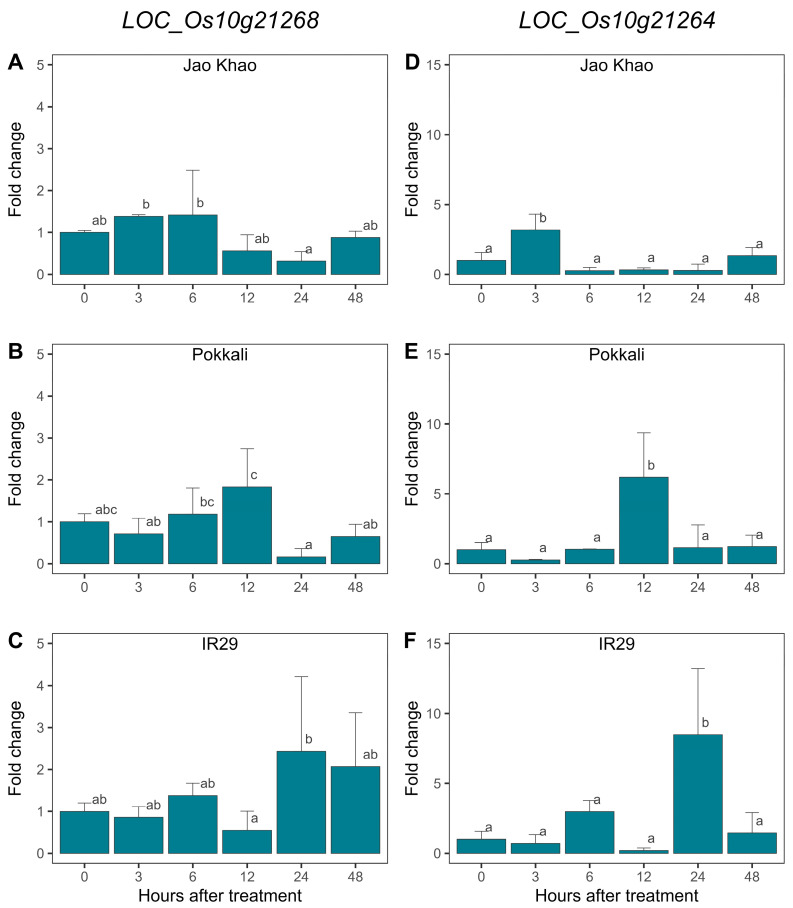
Relative expression levels (fold change) of two genes involved in energy metabolism: *LOC_Os10g21268* and *LOC_Os10g21264* in three varieties: ‘Jao Khao’ (**A**,**D**), ‘Pokkali’ (**B**,**E**), and ‘IR29’ (**C**,**F**) grown under salt stress conditions relative to those under control conditions by RT-qPCR. Data are presented as means ± SD (*n* = 3). Statistical significance was determined using the Duncan multiple range test. Significant differences (*p* ≤ 0.05) are indicated by different letters.

As the significant genes within these modules are primarily involved in energy metabolism processes, such as light reactions, carbon fixation, and ATP synthesis, two genes involved in these processes were selected for expression analysis via RT-qPCR. They included genes encoding RuBisCo (*LOC_Os10g21268*) and ATP synthase (*LOC_Os10g21264*) from the blue module. Their relative expression levels after salt stress treatment for 3, 6, 12, 24, and 48 h were examined in ‘Jao Khao’ and were compared with those of the salt-tolerant (Pokkali) and salt-sensitive (IR29) standard varieties, respectively (Figure 8). Under salt stress conditions, ‘Jao Khao’ exhibited an early upregulation trend of *LOC_Os10g21268* expression at 3 and 6 h after treatment, while ‘Pokkali’ exhibited a delayed upregulation trend at 12 h, followed by ‘IR29’ at 24 and 48 h (Figure 8A–C). For *LOC_Os10g21264*, all three rice varieties significantly exhibited upregulation. ‘Jao Khao’ showed the earliest upregulation at 3 h after treatment, ‘Pokkali’ showed increased expression levels at 12 h, and ‘IR29’ exhibited upregulation at 24 h after treatment (Figure 8D–F).

## 3. Discussion

The salt-tolerant phenotype of the ‘Jao Khao’ Thai rice variety has been extensively documented, with studies demonstrating its ability to maintain high yields even under saline conditions [11,12]. To investigate the molecular mechanisms underlying its salt tolerance, we conducted a comprehensive gene co-expression analysis based on multi-series transcriptome data. ‘Jao Khao’ exhibited remarkable salt-tolerance as evidenced by the nonsignificant changes in CMS, RWC, SPAD index, Fv/Fm, and Pi under saline soil conditions for 9 days. RWC can indicate the water status in plant cells, and salt-tolerant genotypes showed a smaller decrease in RWC compared to the sensitive ones [15]. In Arabidopsis, a higher salt tolerance phenotype is evidenced by an improved capacity to maintain RWC and CMS [16].

Measures of centrality, including DG, CN, BW, and the CC, are used to assess the significance of genes within specific modules. Degree centrality conformed to a power-law distribution, in which genes with fewer connections (low centrality values) outnumbered those with more connections (high centrality values). The power value holds significance because it determines the scale-free topology of the resulting network. A scale-free network is defined by a small number of highly connected nodes (hubs) and numerous nodes with few connections, a structure commonly observed in biological systems [8]. In this work, the distribution of degree centrality (Figure 4A) suggested a decrease in highly connected nodes (approximately connected to 150–450 genes) in the saline-state network, potentially indicating the disruption of connections among genes normally interact under control conditions. Although this resulted in an increase in the number of less connected genes (approximately connected to 1–70 genes), many of them were new connections created under salt stress conditions as detected in all modules except the turquoise module, in which most genes became highly connected under salt stress. Genes deemed less important under normal conditions may play pivotal roles under saline conditions [8].

**Figure 9:**
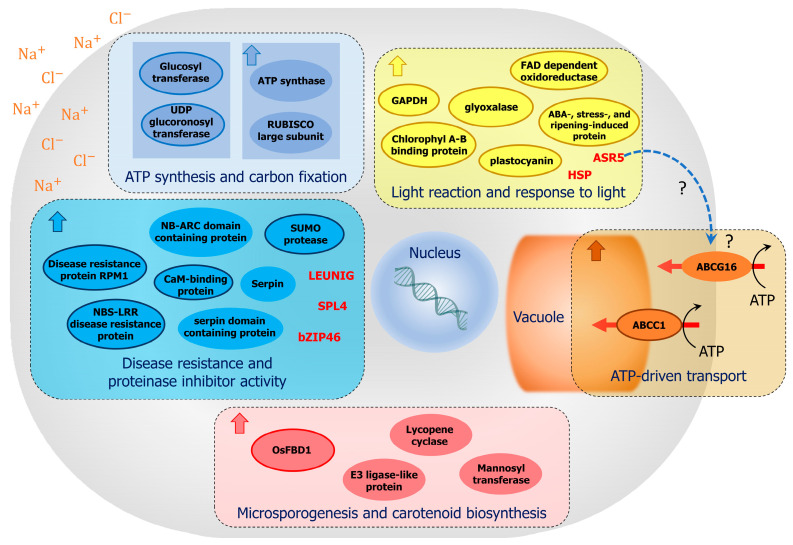
Proposed mechanism of salt stress responses in ‘Jao Khao’ rice as inferred from a comprehensive analysis of time-course transcriptome data. Names bordered by colored lines are key genes. Transcription factors are shown in red letters. GO enrichment analysis revealed several GO terms related to key plant energy metabolism processes, such as light reactions, carbon fixation, and ATP synthesis. Many genes associated with these GO terms exhibited increased expression under salt stress. These enriched processes indicate the importance of maintaining energy production early during salt stress to regulate ion and water uptake and transport. Other enriched GO terms suggested the involvement of ATP-driven transport and the ubiquitination-proteasome pathway in responses to salt stress. Several candidate transcription factors, including *bZIP46*, *SPL4*, *ASR5*, and the transcriptional corepressor LEUNIG, may coordinate these processes. Dash-arrows and question marks suggest potential regulatory relationships.

We compared percentage changes in centrality and genes exhibiting a high percentage change in centrality were identified as key genes, resulting in the clustering of seven modules and the identification of 111 key genes (Table 1). The key genes identified in the modules were validated using publicly available data (Supplementary Table S5) and gene enrichment data (Figure 5 and Supplementary Table S3). Upon examining the validated gene annotations, several key genes potentially involved in the salt-response mechanisms specific to each module were identified (Figure 9). The proposed potential mechanism for each module is described below.

### 3.1. Brown Module

In the brown module, only one key gene, *LOC_Os04g52900*, was identified by DG centrality change, with three combinations of centrality measures (DG, CN, and CC) (Figure 7A). *LOC_Os04g52900* encodes a C-type ATP-binding cassette (ABC) transporter, ABCC1, and was upregulated under salt stress (Figure 9). ABCC1, a member of the ABCC family, participates in the detoxification and reduction of arsenic in rice grains [17]. Another ABCC transporter, OsABCC7, is involved in the root-to-shoot translocation of As(III) [18]. Plant ABCCs can carry out ATP-powered transport of modified toxic compounds (e.g., by conjugation to glutathione or glucose) into the vacuole during the detoxification process [19]. In Arabidopsis, two vacuolar ABCC-type ABC transporters transport ABA glucosyl ester, which is an ABA conjugate that accumulates in the vacuole [20]. In the halotolerant yeast *Debaryomyces hansenii*, vacuolar membrane transporters play major roles in the sequestration of metal ions and some ABCC subfamily members may contribute to salt tolerance [21]. GO enrichment analysis of the brown module identified another ABC transporter gene, *LOC_Os06g51460*, which encodes a G-type ABC transporter (ABCG16). Matsuda et al. [22] reported that RCN1/OsABCG5 functions as a salt-tolerance factor by affecting the balance of sodium and potassium, possibly via the control of SKC1/OsHKT1;5 in the shoots.

### 3.2. Turquoise Module

The turquoise module contained numerous genes, including 59 key genes that exhibited high centrality (Figure 7B). Among these, 43 genes were detected only in the saline-state network. The genes primarily enriched in this module were associated with GO terms related to disease resistance and serine-type endopeptidase inhibitor activity and were upregulated early during salt stress (Figure 9). One of the enriched GO terms was ADP binding, which included *LOC_Os09g14100* (NB-LRR, disease resistance protein RPS2), *LOC_Os11g10610* (NBS-LRR disease resistance protein), *LOC_Os11g11790* (NBS-LRR type disease resistance protein), *LOC_Os11g11810* (NBS-LRR disease resistance protein), and *LOC_Os11g11770* (disease resistance protein RPM1, similar to the NBS-LRR protein fragment), all of which were key genes. The NBS-LRR family, which is characterized by the presence of a nucleotide-binding site (NBS) and leucine-rich repeat (LRR) domains and may act as receptors in immune and non-immune responses to biotic and abiotic stresses (drought and salt stresses), was prevalent in the turquoise module, suggesting potential signaling overlap between these responses [23]. The genes in this module associated with the enriched GO term proteinase inhibitor activity included *LOC_Os11g11760* (OsSRP-PSG, serpin domain-containing protein), *LOC_Os11g12410* (OsSRP-GAA, serpin domain-containing protein), *LOC_Os11g12420* (serpin-Z6B), *LOC_Os11g13530* (serpin-Z2A), and *LOC_Os11g13540* (serpin-Z2B), all of which were serine protease inhibitors in the serpin superfamily.

Additional key genes in this module included *LOC_Os11g44340* and *LOC_Os11g44170*, which encode calmodulin-binding proteins. In eukaryotes, calmodulin is an intracellular Ca^2+^-binding protein that interacts with more than 300 target proteins and peptides [24]. In rice, it plays various roles as a signal transducer in the response to salt stress [25,26]. Other key genes were *LOC_Os01g25370* (SUMO protease), which is involved in SUMOylation, resulting in early flowering and decreased plant height in rice [27], and *LOC_Os01g42350*, *LOC_Os11g05700*, and *LOC_Os01g42370*, encoding transporters including pleiotropic drug resistance proteins and ABC transporter family proteins, which are involved in abiotic and biotic stresses, such as iron deficiency stress [28].

Key genes encoding transcription factors in this module included *LOC_Os02g07780* (SPL4-SBP-box gene family member), which encodes the SQUAMOSA promoter-binding protein domain, a transcription factor that regulates grain size [29], *LOC_Os01g42260* (LUGL, transcriptional corepressor LEUNIG), which influences floral development by modulating auxin levels [30], and *LOC_Os06g10880* (bZIP46 transcription factor), which is positively regulated by ABA signaling and drought stress in rice [31].

### 3.3. Yellow Module

In the yellow module, 13 key genes were identified (Figure 7C). Among them, eight genes were specific to the saline-state network. Genes in this module were enriched in the GO terms light reactions and response to light. The key genes associated with the enriched GO terms included *LOC_Os03g39610* (LHCB, chlorophyll A-B binding protein), which is involved in photosynthesis, light harvesting, and the response to light [32] The expression of *LOC_Os03g39610* and several other enriched genes associated with photosynthesis and light harvesting initially decreased, but later increased (Figure 9). A similar expression pattern was also observed for *LOC_Os03g03720* (glyceraldehyde-3-phosphate dehydrogenase, GAPDH), which is a potential candidate gene involved in rice tolerance to low light stress and is related to aromatic traits.

Another key gene in this module was *LOC_Os11g06720* gene (ASR5, abscisic acid-, stress-, and ripening-induced protein), which encodes a transcription factor that plays a role in aluminum tolerance by binding STAR1, which encodes an ABC transporter [33]. ASR5 also plays an important role in the drought stress response by regulating ABA biosynthesis, facilitating stomatal closure, and acts as chaperone-like protein to prevent the inactivation of drought stress-related proteins [33,34].

Other key genes were *LOC_Os07g34570* (DR8, FAD-dependent oxidoreductase domain containing protein), which is involved in disease resistance [35,36], *LOC_Os06g01210* (PC, plastocyanin, chloroplast precursor), which functions in electron transport [37], *LOC_Os01g10400* (MT-3a, metallothionein-like protein), which provides tolerance to salinity, drought, and heavy metal stress in rice [38], *LOC_Os08g09250* (GLYI11.2, glyoxalase family protein), which is related to plant stress adaptation [39], and *LOC_Os08g39300* (SGAT, serine:glyoxylate aminotransferase), which controls crucial photorespiratory enzymes in rice at both the transcriptional and post-translational (phosphorylation) levels [40].

### 3.4. Blue Module

Seventeen of the 18 key genes in the blue module were present only in the saline-state network (Figure 7D). The main GO terms enriched in this module were hexosyltransferase activity, ATP synthase activity, and carbon fixation. The genes associated with hexosyltransferase activity were all key genes whose expression did not significantly change under salt stress (Figure 9), including *LOC_Os05g12450* (UGT, hydroquinone glucosyltransferase), which is triggered by both biotic and abiotic stresses [41], *LOC_Os03g11350* (UDP-glucoronosyl and UDP-glucosyl transferase), and *LOC_Os03g55020* (UDP-glucoronosyl and UDP-glucosyl transferase domain containing protein), which participates in pathogen defense responses [42].

The genes associated with ATP synthase activity and carbon fixation were upregulated under salt stress, unlike the key genes in this module. These genes included *LOC_Os12g10570* (ATP synthase subunit beta), which is involved in carbon and nitrogen metabolism and upregulated under salt stress, *LOC_Os10g21264* (ATP synthase epsilon chain), which was identified by genome-wide association in rice exposed to short-term exposure to salt stress [43], and *LOC_Os10g21268* and *Os01g0791033*, which encode the large subunits of RuBisCo, the key enzyme in carbon fixation and photorespiration. No other genes encoding other subunits ATP synthase or RubisCo were identified.

### 3.5. Red Module

In the red module (Figure 7E), only one key gene was identified, *LOC_Os01g39670* (FBD1), with two combinations of centrality measures (DG and CC). Genes associated with the enriched GO term microsporogenesis included the key gene FBD1 and *LOC_Os01g39680* (small ubiquitin-like modifier (SUMO) E3 ligase-like protein) (Figure 9). FBD1, an F-box and fibrin-binding domain (FBD) protein involved in floral transition, panicle development, and seed formation [44]. The F-box protein is part of two-gene male sterility systems involved in male fertility in indica–japonica hybrids [45,46]. *LOC_Os01g39960* (lycopene epsilon cyclase, chloroplast precursor) and *LOC_Os01g39810* (Alg9-like mannosyltransferase protein) were associated with the carotenoid biosynthetic process and GPI anchor biosynthetic process, respectively.

Expression of the two genes involved in energy metabolism, RuBisCo and ATP synthase, in ‘Jao Khao’ was early upregulated under salt stress conditions. ATP synthase is the enzyme that utilizes electrochemical energy to power the synthesis of ATP, a key process involved in active ion transport. In plants, it has been reported that ATP synthase is overexpressed in response to abiotic stresses [47,48]. Wei et al. [49] suggested that increased ATP production in cells could improve plant tolerance to salt stress. Wang et al. [50] reported that *LOC_Os10g21264* was one of the most downregulated DEGs in the *bglu6* mutant, which is involved in ABA recycling in rice under drought conditions. In this study, two ATP synthase genes were co-expressed in the same module. ATP synthase typically consists of two main parts (F_o_ and F_1_) that work together. LOC_Os10g21264 (epsilon subunit), containing a GO term enriched in this study, was selected for validation using RT-qPCR, and the results showed that it was upregulation early. Together with LOC_Os12g10570 (beta subunit), they were the only two ATP synthase genes that showed upregulation in ‘Jao Khao’ during salt stress. This is similar to a previous study on salt-stress response in *Triticum aestivum*, in which few genes encoding ATP synthase F_1_ subunits (beta and delta) were upregulated during salt stress; however, genes encoding other F_1_ subunits did not show upregulation [51]. Based on both studies, other regulatory mechanisms of gene expression, that is, translational and posttranslational regulation for genes that encode other subunits of ATP synthases may play a role in response to salt stress without altering the transcript levels. Even with an upregulation of transcript levels of only few genes encoding F_1_ subunits, these mechanisms may result in better maintenance of photosynthetic activity and energy production during salt stress as observed in ‘Jao Khao’ variety in this study. Additionally, in a previous study, the overexpression of a gene encoding a component of the F_o_ part of the rice mitochondrial ATP synthase was shown to increase salt tolerance; however, the transcript levels of the genes encoding the other mitochondrial F_1_F_o_-ATPase subunits were mostly not affected by salt stress. This study showed that an increase in the expression of one gene encoding a single component of the ATP synthase could lead to the difference in salt tolerance ability [48]. In this study, the early upregulation of ATP synthase (*LOC_Os10g21264*) in ‘Jao Khao’ aligns well with its ability to maintain light energy absorption, as indicated by maximum PSII efficiency (Fv/Fm), Performance index (Pi), and survival under saline stress.

Based on our data of gene co-expression network, LOC_Os10g21268 (RuBisCo large subunit) was detected and matched with enriched GO terms; therefore, we selected this gene for validation. RuBisCo is a multi-subunit enzyme that functions as a complex. It consists of eight large (RbcL) and eight small (RbcS) subunits, which come together to form a functional enzyme. There are multiple annotated RuBisCo large chain and small chain precursor genes in the rice genome with LOC_Os10g21268 that exhibit very high expression levels in leaf (based on RNA-Seq data in the Rice Genome Annotation Project database). The small subunit gene, LOC_Os12g19381, which also exhibited high expression levels in leaf, showed a trend of upregulation under salt stress in this study; however, the difference was not statistically significant, and it was not picked up by the gene co-expression network analysis. For Rubisco to work effectively in catalyzing CO_2_-fixing reaction during photosynthesis, all subunits must be correctly assembled and function together. However, in a previous study in rice, overexpression of a RuBisCo large subunit gene alone was found to improve drought tolerance [52].

While these findings underscore the significance of these two genes in energy production under salinity stress, it is important to note that this discovery is based on a single rice variety. This genetic feature might be specific to certain genotypes and not necessarily present in all rice varieties. In addition, salt tolerance is a complex trait regulated by multiple genes and various cellular processes. The identified genes likely contribute to a portion of this trait, interacting with other genes within the plant’s genetic backgrounds. The interplay between these genes, each with its own variations, contributes to the diverse salt tolerance observed across different rice varieties.

Overall, GO enrichment analysis revealed gene enrichment in several GO terms involved in central plant energy metabolism, including light reactions, carbon fixation, and ATP synthesis, under saline conditions, which are in agreement with the ability of ‘Jao Khao’ in maintaining the SPAD index, Fv/Fm, and Pi during the 9-day of salt stress. Most of the genes associated with these GO terms were upregulated under saline conditions. These enriched processes suggest the importance of maintaining energy production early during salt stress for the regulation of ion and water uptake and transport [53]. Further, the results suggested the involvement of ATP-driven transport and the ubiquitination-proteasome pathway in the response of ‘Jao Khao’ to salt stress. Several candidate transcription factors may coordinate these processes, including *bZIP46*, *SPL4*, *ASR5*, and the transcriptional corepressor LEUNIG. Our findings suggest that these genes may play crucial roles in the response of rice to salt stress.

## 4. Materials and Methods

### 4.1. Phenotyping of Rice under Soil-Salt Treatment

‘Jao Khao’ seeds were germinated in water for 10 days, then transplanted into clay soil with one seedling in a 40 cm^3^ pot, supplemented with 16-16-16 fertilizer, for two weeks in a greenhouse under ambient conditions. Two growth conditions were separately established in each tray by soaking in 15 L tap water: normal conditions, in which no salt was applied, and saline conditions, in which the soil was treated with 160 mM NaCl, in a complete randomized design (CRD) with three replicates. This procedure was used for phenotyping, RNA sequencing, and RT-qPCR of separate individual plants after treatment with salt stress at indicated timepoints. At 0, 3, 6, and 9 days after stress exposure, phenotypic characteristics, including shoot and root fresh and dry weights, tiller number, the standard salt injury evaluation system (SES) score, cell membrane stability (CMS), and relative water content (RWC). We conducted additional experiments using the same protocol as described earlier by growing ‘Jao Khao’ and comparing it with that of ‘Pokkali’ (a standard salt-tolerant variety) and ‘IR29’ (a standard salt-sensitive variety) in a CRD with three replications for each time point to determine CMS, RWC, SPAD index, and chlorophyll fluorescence (Fv/Fm and performance index). The experimental methodologies followed the procedures outlined by Kojonna, et al. [16], and Punchkhon, et al. [54]. Statistical significance was assessed using the Duncan multiple range test, with significance defined as *p* ≤ 0.05.

### 4.2. RNA Extraction, Library Preparation, and Sequencing

Fully expanded leaves were collected from three replicates under normal and saline (160 mM NaCl) soil conditions at 0, 3, 6, 12, 24, and 48 h following salt treatment. The leaves were immediately frozen in liquid nitrogen and stored at −80 °C. Total RNA was extracted from the leaves using PureLink™ Plant RNA Purification Reagent (Thermo Fisher Scientific, Waltham, MA, USA), and genomic DNA was removed using DNase I (RNase-free; New England Biolabs, Ipswich, MA, USA). The RNA quality was assessed using an Agilent 2100 bioanalyzer (Agilent, Santa Clara, CA, USA) and RNA concentration was determined using a DeNovix fluorometer (DeNovix, Wilmington, DE, USA). Individually indexed strand-specific RNA-sequencing libraries of all samples were prepared using QIAseq FastSelect–rRNA Plant and QIAseq Stranded mRNA Library kits (Qiagen, Hilden, Germany). The indexed libraries were pooled in equimolar quantities and subjected to cluster generation and 150-nucleotide paired-end sequencing on an Illumina HiSeq sequencer (Illumina, San Diego, CA, USA). In total, 36 cDNA libraries were sequenced.

### 4.3. Quantification of Gene Expression by RT-qPCR

The youngest fully expanded leaves of ‘Jao Khao’, ‘Pokkali’, and ‘IR29’ were collected at different time points as previously described. RT-qPCR was conducted following the methodology described by Chinpongpanich et al. [25], using three technical replications and at least two biological replicates to assess the gene expression levels of *LOC_Os10g21268* and *LOC_Os10g21264*. The oligonucleotide primers used were as follows: *LOC_Os10g21268*: forward primer—5′ TTCCGAGTAACTCCTCAGCC and reverse primer—5′ GATTATCCTCCCCAACAACGG; and *LOC_Os10g21264*: forward primer—5′ GCCCGCCTTTATCGAGTTAG and reverse primer—5′ TCCCCTACTCCGCCAAATAC. EF-1-α (*LOC_Os03g08010*) was used as internal control with the following primers: forward—5′ ATGGTTGTGGAGACCTTC and reverse—5′ TCACCTTGGCACCGGTTG.

### 4.4. Data Preprocessing and Screening

**Figure 10:**
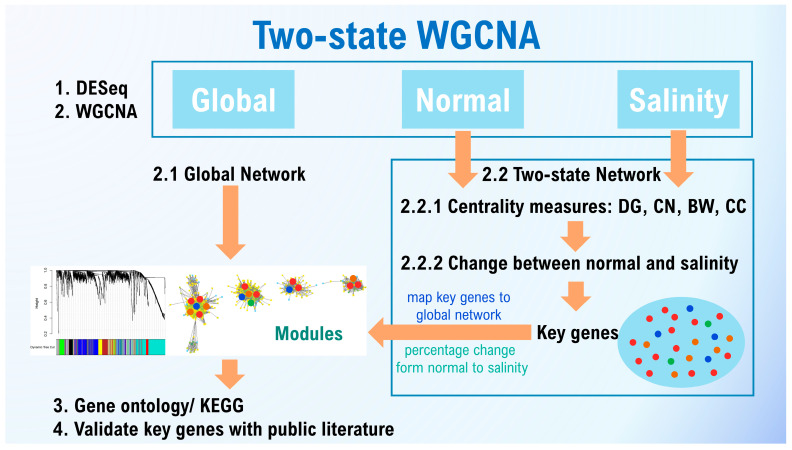
Diagram illustrating the co-expression network analysis pipeline used in the present study.

The raw reads were subjected to quality control assessment using the FASTQC software (version 0.12.0) and trimmed using Trimmometic-0.39 (https://github.com/usadellab/Trimmomatic/ (accessed on 26 February 2024)). Then, the reads were aligned to the rice reference genome IRGSP-1.0 obtained from the EnsemblPlants database (https://plants.ensembl.org/index.html (accessed on 26 February 2024)) using HISAT2 (https://daehwankimlab.github.io/hisat2/ (accessed on 26 February 2024)). The alignment results in SAM format were converted to the BAM format and indexed using SAMtools (version 1.17) [55]. The aligned reads were quantified using HTSeq-count (https://htseq.readthedocs.io/en/release_0.11.1/count.html (accessed on 26 February 2024)), with the genomic features provided in a GTF file (https://plants.ensembl.org/index.html (accessed on 26 February 2024)). The 36 raw read count datasets were normalized by division by the size factors. To this end, we calculated the variance stabilizing transformation of the read counts of each gene using the ‘varianceStabilizingTransformation’ function in DESeq2 [56]. Genes with low variation were filtered out using the ‘genefilter’ package [57]. Genes passing the 95th percentile threshold were used in the WGCNA pipeline (Figure 10).

### 4.5. WGCNA

We used WGCNA to construct global, normal-state, and saline-state networks to compare the co-functional genes under normal and saline conditions, following the pipeline outlined in Sonsungsan et al. [8] and Suratanee et al. [58] (Figure 10). The transcriptome data were normalized using DESeq2 [56], and genes with high variation were selected. Next, we constructed three WGCNs: one for the global state (using all 36 libraries), one for the normal state (using 18 libraries), and one for the saline state (using 18 libraries). The scale-free topology was assessed to determine the optimal soft threshold (power, β) for calculating the adjacency matrix based on the Pearson correlation of pairwise genes. A power of 10 was selected as the minimum power at which the scale-free topology fit index curve flattens, achieving a high value (*R*^2^ = 0.974). The normal- and saline-state networks obtained were used to qualify the level of importance of the genes based on various centrality measures.

### 4.6. Centrality Measurement and Identification of Key Hub Genes

In biological systems, genes exert distinct functions while intricately interacting with each other [59]. A GCN represents connections among entities [60], in which each node represents a gene and the edges represent connections between genes. We analyzed biological networks focusing on key centrality measures such as degree, betweenness, closeness, and the clustering coefficient. Genes exhibiting high centrality properties were denoted as ‘hub genes’ [61]. The centrality measures, as described by Sonsungsan et al. [7], can be summarized as follows (for details, refer to Supplementary Figures S5–S7).

The degree (DG) of a node is determined by counting the edges connected to it [62]. A high DG indicates significant centrality, implying that the corresponding gene has numerous interactions. Closeness centrality (CN) reflects the average distance of the shortest path from a node to all others in the network, with higher values indicating greater centrality [63]. CN values range from 0 to 1. Betweenness centrality (BW) quantifies the proportion of shortest paths that pass through the node, with a higher BW facilitating network-wide communication. BW helps identify pivotal ‘bridge-spanning’ nodes that serve as key connectors within the network [64]. The BW of each node ranges from 0 to 1 [7]. The clustering coefficient (CC) measures the proportion of neighbors that are also neighbors of each other. It provides insight into the local network structure, revealing how densely interconnected the immediate neighbors of a node are [7,58,60].

To prepare the data for calculating centrality, the three networks were exported from WGCNA (version 1.73) using the ‘exportNetworkToCytoscape’ function, and the threshold for the lowest possible correlation was set to 0.1. Subsequently, the ‘Analyze Network’ function in Cytoscape (version 3.10.0) [65] was used to calculate centralities including DG, BW, CN, and CC. The network property was generated using the same function, which included number of nodes, number of edges, average degree, diameter, average clustering coefficient and number of connections per node. All four centrality measures were used to assess the changes from the normal-state network to the saline-state network. Two criteria were employed to identify key hub genes: first, only genes loaded into the global network, and second, the percentage of centrality changes from less important genes (low scores) in the normal state to genes with high importance (high scores) in the saline state [7]. The key genes were selected from those exhibiting the percentage change in DG centrality that surpassed 80%.

### 4.7. Module Detection and Functional and Pathway Enrichment Analyses

Modules in the global co-expression network were detected using WGCNA [4] by calculating the dissimilarity of the topological overlap matrix. The relationship between the modules and the time-course of gene expression was analyzed via WGCNA. Next, the modules were examined for related functions or pathways. We used public annotation data from the Rice Annotation Project Database (IRGSP-1.0-2024-01-11) and OryzaBase (https://shigen.nig.ac.jp/rice/oryzabase/ (accessed on 26 February 2024)) to conduct a Gene Ontology (GO) enrichment analysis, classifying the genes in each global network module based on their function into biological process (BP), cellular component (CC), and molecular function (MF) categories, using the clusterProfiler R function [14]. Additionally, we performed a pathway enrichment analysis by downloading gene annotations from the Kyoto Encyclopedia of Genes and Genomes (KEGG) (https://www.kegg.jp/ (accessed on 26 February 2024)).

## 5. Conclusions

We compared transcriptome data from ‘Jao Khao’ rice seedlings grown under normal and saline conditions at multiple time points to identify key hub genes associated with salt tolerance. From the 111 genes selected based on centrality measures from a pool of 1950 highly variable genes by two-state WGCN and GO enrichment analysis, the identified genes involved in ATP-driven transport, light reactions and response to light, ATP synthesis, carbon fixation, disease resistance, and proteinase inhibitor activity were identified as important components of the salt-stress response in rice. Early upregulation of these genes suggests their importance in response to this stressor. Expression of two genes involved in energy metabolism (RuBisCo and ATP synthase) was upregulated early in the salt-tolerant variety, ‘Jao Khao.’ This indicates that it is important for the plant to manage energy for their survival in the initial phase of salt stress and the processes and key genes identified in this study potentially play important roles. These findings suggest that manipulation of the energy metabolism should be the focus in plant resistance breeding effort.

## Figures and Tables

**Table 1:** Modules and key genes.

Module	Grey	Green	Turquoise	Yellow	Red	Blue	Brown	Sum
Total genes	362	59	610	113	48	567	191	1950
Key genes	14	5	59	13	1	18	1	111
Percentage	3.9	8.5	9.7	11.5	2.1	3.2	0.5	5.7

**Table 2:** Network properties of the three co-expression networks.

Network Properties	Global Network	Normal-State Network	Salinity-State Network
Number of nodes	909	1204	1351
Number of edges	42,543	59,749	54,090
Average degree	157.002	206.420	120.038
Diameter	7	9	16
Average clustering coefficient	0.845	0.831	0.806
Number of connections per node	28	52	21

## Data Availability

The authors confirm that data supporting the findings of this study are available in the article and its Supplementary Materials (Supplementary Tables S1–S5, Supplementary Figures S1–S7). The exome sequence raw data is available through direct contact with the corresponding author.

## Supplementary Materials

The following supporting information can be downloaded at: https://www.mdpi.com/article/10.3390/ijms252011086/s1.
