# Supplementary material for: Profiling of Key Hub Genes Using a Two-State Weighted Gene Co-Expression Network of ‘Jao Khao’ Rice under Soil Salinity Stress Based on Time-Series Transcriptome Data
Source: Int J Mol Sci. 2024 Oct 16;25(20):11086. doi: 10.3390/ijms252011086 (PMC11508143; doi:10.3390/ijms252011086)
Supplement: Supplementary file 1 [file ijms-25-11086-s001.zip › Supplementary_materials_Figure_S1-S7.pdf]

International Journal of *Molecular Sciences*

Supplementary Materials

Article

Profiling of Key Hub Genes Using a Two-State Weighted Gene Co-Expression Network of 'Jao Khao' Rice under Soil Salinity Stress Based on Time-Series Transcriptome Data

Prasit Khunsanit, Kitiporn Plaimas, Supachitra Chadchawan, and Teerapong Buaboocha *

*** Correspondence:** teerapong.b@chula.ac.th

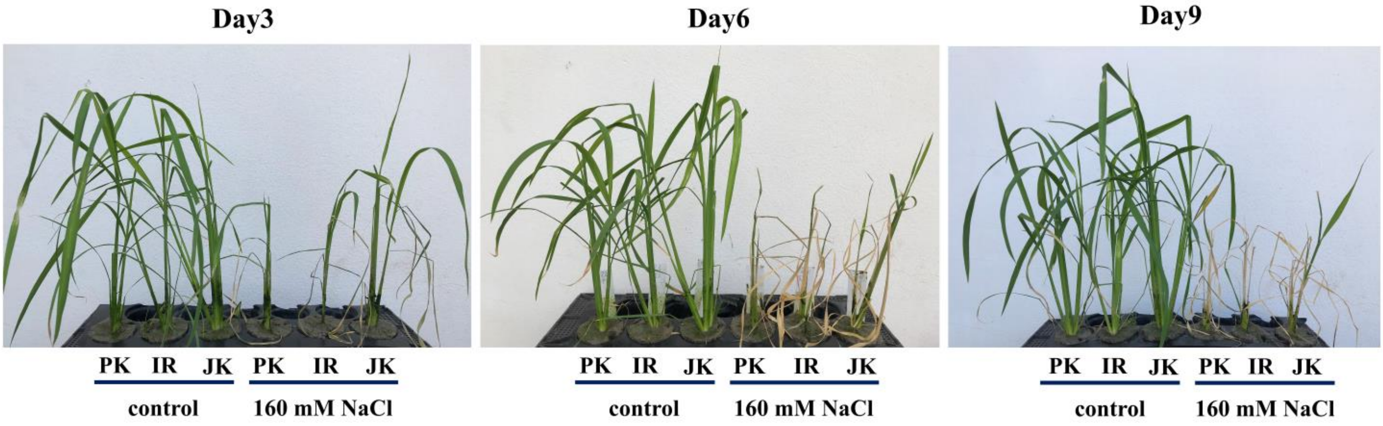

Supplementary Figure S1. Four-week-old seedlings of three rice varieties treated under control and salt conditions for 9 days: PK (Pokkali), IR (IR29), and JK (Jao Khao).

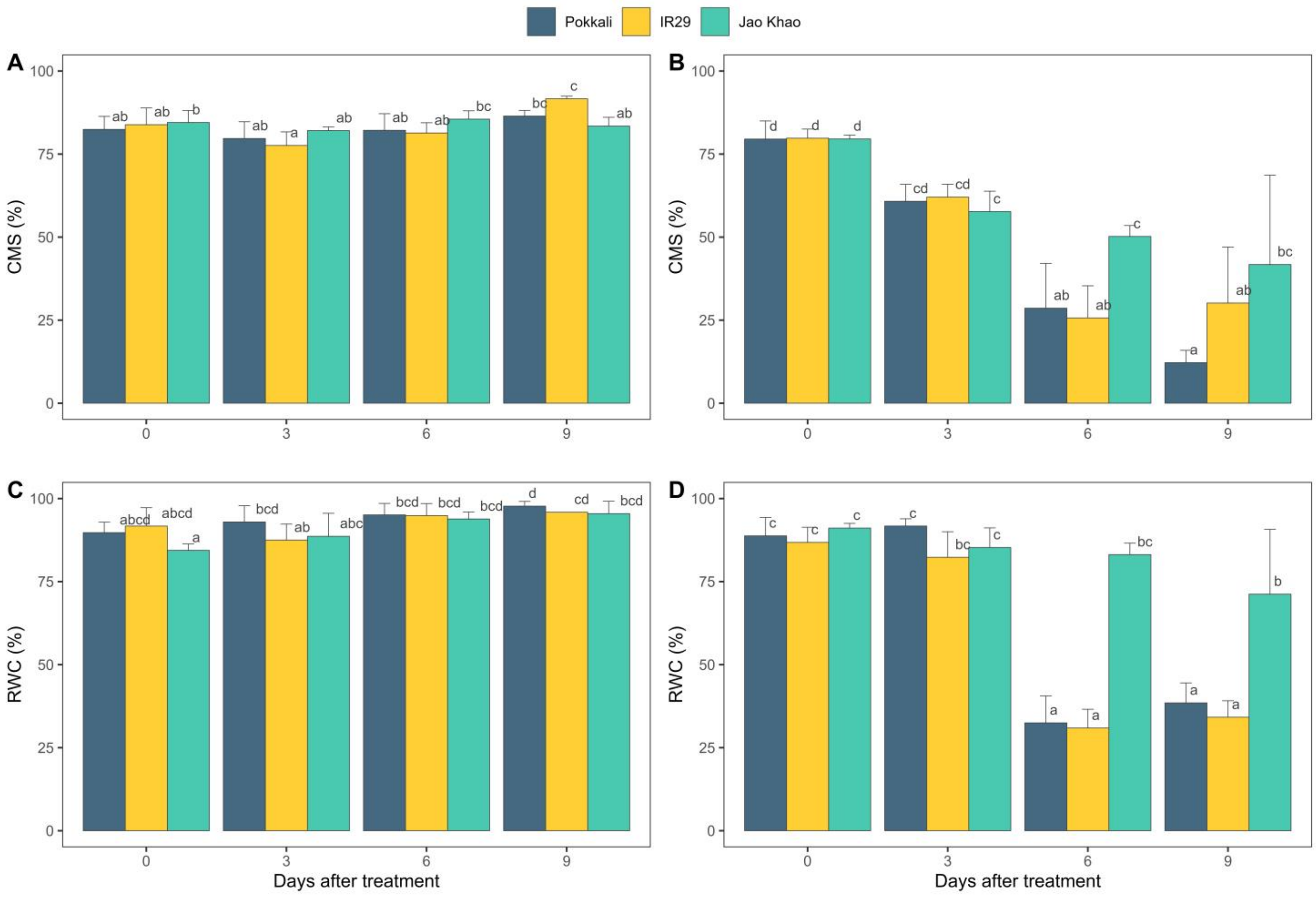

Supplementary Figure S2. Cell membrane stability (CMS) and relative water content (RWC) of three rice varieties in control (**A, C**) and salt stress conditions (**B, D**) over 9 days. Data are presented as means ± SD (n = 3). Statistical significance was determined using the Duncan multiple range test. Significant differences ($P \leq 0.05$) are indicated by different letters.

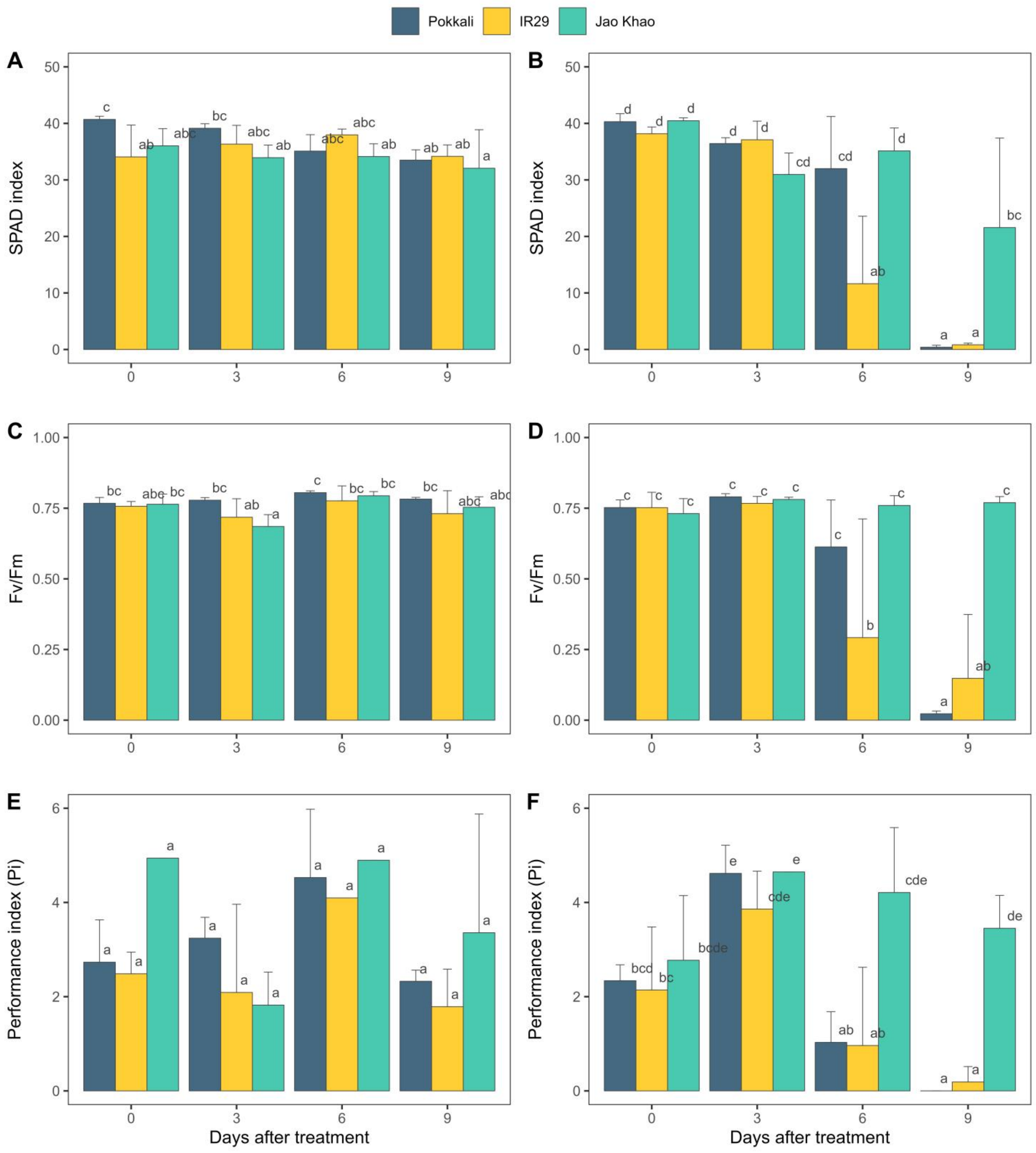

Supplementary Figure S3. Leaf greenness and chlorophyll fluorescence parameters including SPAD index, maximum PSII efficiency (Fv/Fm), and Performance index (Pi) of three rice varieties in control (**A, C, E**) and salt conditions (**B, D, F**) over 9 days. Data are presented as means ± SD (n = 3). Statistical significance was determined using the Duncan multiple range test. Significant differences ($P \leq 0.05$) are indicated by different letters.

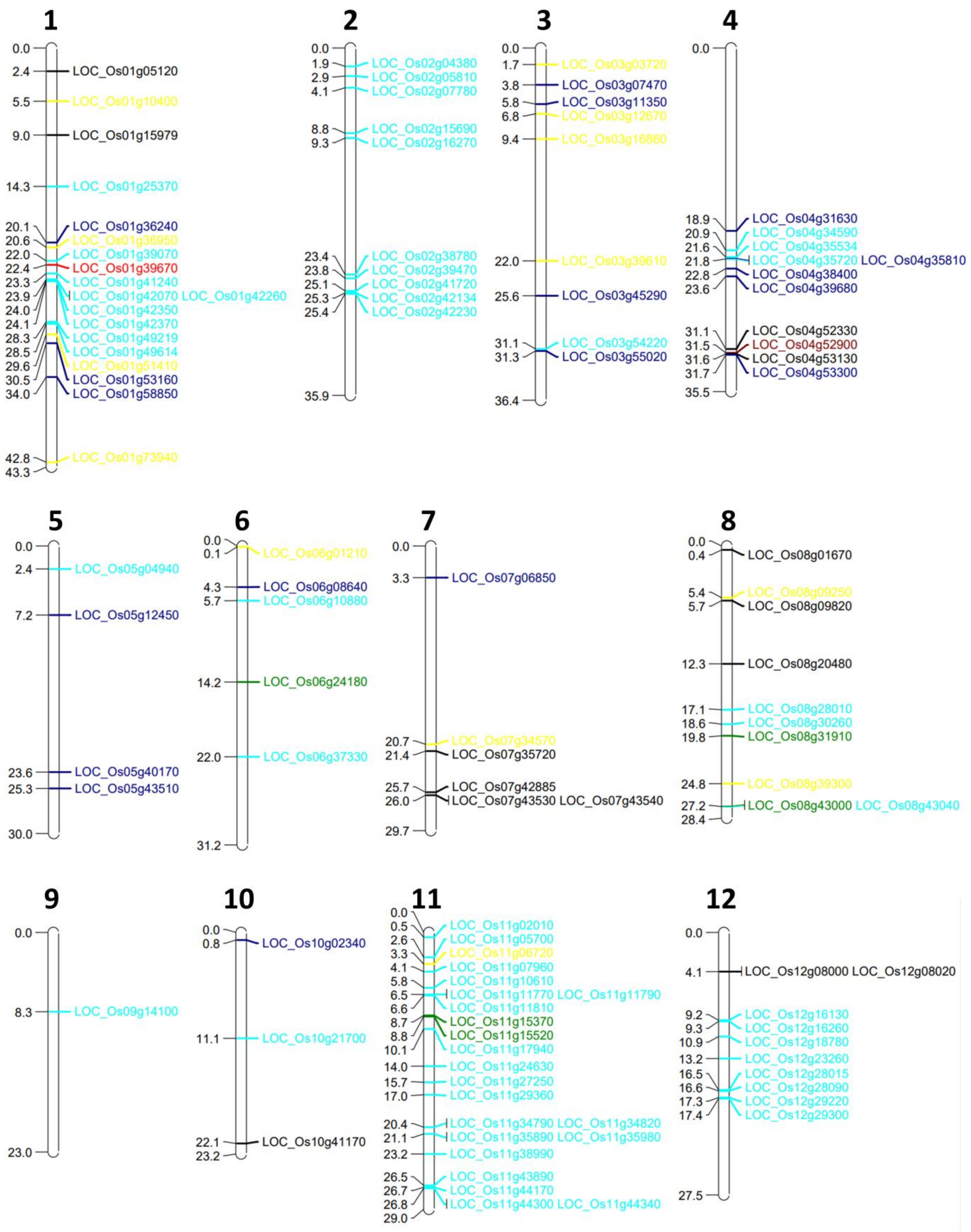

Supplementary Figure S4. Distribution of the 111 key genes identified in the modules (grey [14 genes], green [five genes], turquoise [59 genes], yellow [13 genes] red [one gene], blue [18 genes], and brown [one gene]) across the 12 rice chromosomes.

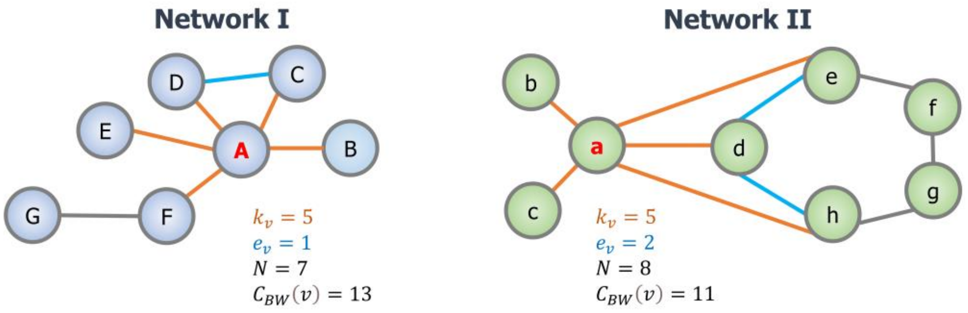

Degree

$C_{DG}(v) = deg(v)$

Where $deg(v)$ = no. of direct connected node of v

Closeness

$C_{CN}(v) = 1/avg(L(u,v))$

Where $L(u,v)$ = shortest path between u, v

Betweenness

$$C_{BW}(v) = \sum_{s \neq n \neq t} (\sigma_{st}(v)/\sigma_{st})$$

Where s and t are node differnce from v

σ_{st} = no. of shortest path from s to t

$\sigma_{st}(v)$ = no. of shortest path from s to t pass v

Normalized $C_{BW}(v)$by dividing with $(N-1)(N-2)/2$

Where N = total no. of nodes of network v

Clustering coefficient

$$CC_v(v) = 2e_v/(k_v(k_v - 1))$$

Where k_v = node degree of v

e_v =no. of edge that connect the immediate neighbors of a node v

Centrality	Description	Considering	Network I	Network II
Degree	Number of direct edges	A and a	5	5
Closeness	Centered with shortest path	A and a	1/1=1	1/1.13=0.88
Betweenness	Shortest path between node s,t	A and a	13/15=0.87	11/21=0.52
Clustering Coefficient	Interconnected edges	A and a	2x1/(5x(5-1))=0.1	2x2/(5x(5-1))=0.2

Summary of centrality measurement

Degree:	A = a
Closeness:	A > a
Betweenness:	A > a
Clustering Coefficient:	A < a

Supplementary Figure S5. Comparison of mathematics and examples between two networks based on centrality measurement, including degree (DG), closeness (CN), betweenness (BW), and clustering coefficient (CC).

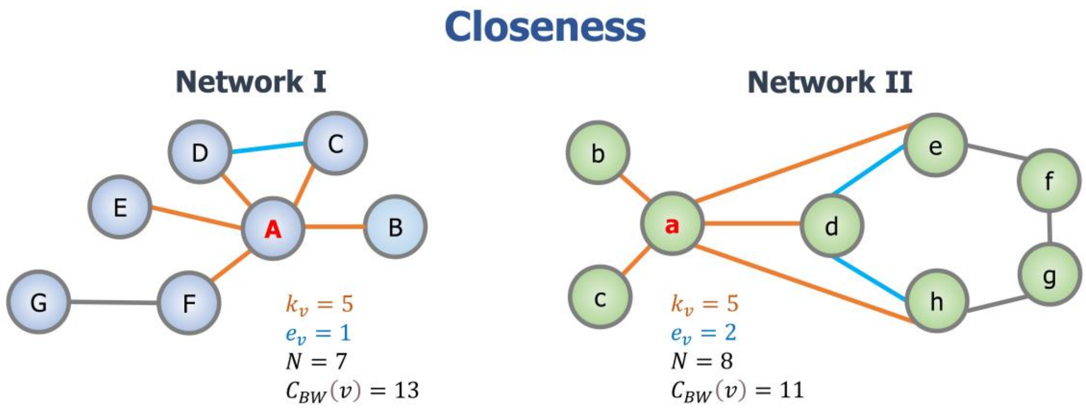

	A	B	C	D	E	F	G	sum	avg	$C_{CN}(v)$
**A**	0	1	1	1	1	1	2	7	1.0	1/1.0=1.00
B	1	0	2	2	2	2	3	12	1.7	1/1.7=0.58
C	1	2	0	1	2	2	3	11	1.6	1/1.6=0.63
D	1	2	1	0	2	2	3	11	1.6	1/1.6=0.63
E	1	2	2	2	0	2	3	12	1.7	1/1.7=0.58
F	1	2	2	2	2	0	1	10	1.4	1/1.4=0.71
G	2	3	3	3	3	1	0	15	2.1	1/2.1=0.47

	a	b	c	d	e	f	g	h	sum	avg	$C_{CN}(v)$
**a**	0	1	1	1	1	2	2	1	9	1.13	1/1.13=0.88
b	1	0	2	2	2	3	3	2	15	1.86	1/1.86=0.54
c	1	2	0	2	2	3	3	2	15	1.86	1/1.86=0.54
d	1	2	2	0	1	2	2	1	11	1.38	1/1.38=0.72
e	1	2	2	1	0	1	2	2	11	1.38	1/1.38=0.72
f	2	3	3	2	1	0	1	2	14	1.75	1/1.75=0.57
g	2	3	3	2	2	1	0	1	14	1.75	1/1.75=0.57
h	1	2	2	1	2	2	1	0	11	1.38	1/1.38=0.72

Supplementary Figure S6. The closeness measurement is calculated using the equation $C_{CN}(v) = 1/avg(L(u,v))$, where closeness measures the shortest path from node u to node v. In this example, considering nodes A and a, the shortest paths from A or a to other nodes were calculated, and the average (avg) was subsequently determined. Subsequently, the reciprocal of the average is taken. Closeness values range between 0 and 1, with larger values indicating greater centrality importance. In this case, $C_{CN}(A) = 1$ and $C_{CN}(a) = 0.88$, indicating that node A is more important than node a.

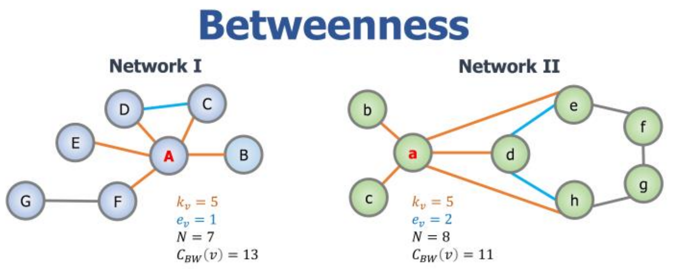

		σ_{st}	$\sigma_{st}(v)$	$(\sigma_{st}\,(v)/\sigma_{st})$
1	(B,C)	1	1	1
2	(B,D)	1	1	1
3	(B,E)	1	1	1
4	(B,F)	1	1	1
5	(B,G)	1	1	1
6	(C,D)	1	0	0
7	(C,E)	1	1	1
8	(C,F)	1	1	1
9	(C,G)	1	1	1
10	(D,E)	1	1	1
11	(D,F)	1	1	1
12	(D,G)	1	1	1
13	(E,F)	1	1	1
14	(E,G)	1	1	1
15	(F,G)	1	0	0
			sum	13

(N-1)(N-2)/2

(7-1)(7-2)/2=15

13/15=0.87

		σ_{st}	$\sigma_{st}(v)$	$(\sigma_{st}\,(v)/\sigma_{st})$
1	(b,c)	1	1	1
2	(b,d)	1	1	1
3	(b,e)	1	1	1
4	(b,f)	1	1	1
5	(b,g)	1	1	1
6	(b,h)	1	1	1
7	(c,d)	1	1	1
8	(c,e)	1	1	1
9	(c,f)	1	1	1
10	(c,g)	1	1	1
11	(c,h)	1	1	1
12	(d,e)	1	0	0
13	(d,f)	1	0	0
14	(d,g)	1	0	0
15	(d,h)	1	0	0
16	(e,f)	1	0	0
17	(e,g)	1	0	0
18	(e,h)	1	0	0
19	(f,g)	1	0	0
20	(f,h)	1	0	0
21	(g,h)	1	0	0
			sum	11

(N-1)(N-2)/2

(8-1)(8-2)/2=21

11/21=0.52

Supplementary Figure S7. The betweenness measurement is calculated using the equation $C_{BW}(v) = \sum_{s\neq n\neq t}(\sigma_{st}\,(v)/\sigma_{st})$, where $\sigma_{st}(v)$ represents the short path from node s to node t passing through node v and σ_{st} is the short path from s to t, allowing for the possibility of passing through v. To calculate this, the process is performed for all pairs of nodes except node v. The values $(\sigma_{st}\,(v)/\sigma_{st})$ for all pairs are then summed. Next, this sum is normalized by dividing it by $(N-1)(N-2)/2$, where N is the total number of nodes in network v. Betweenness values range between 0 and 1, with higher values indicating significant centrality. In this case, $C_{BW}(A) = 0.87$ and $C_{BW}(a)$ =0.52. Hence, node A is more important than node a is, suggesting that node A plays a crucial role as key connecting bridge in network I.
