# Supplementary material for: Profiling of Key Hub Genes Using a Two-State Weighted Gene Co-Expression Network of ‘Jao Khao’ Rice under Soil Salinity Stress Based on Time-Series Transcriptome Data
Source: Int J Mol Sci. 2024 Oct 16;25(20):11086. doi: 10.3390/ijms252011086 (PMC11508143; doi:10.3390/ijms252011086)
Supplement: Supplementary file 1 [file ijms-25-11086-s001.zip › Supplementary_materials_Table_S5.pdf]

International Journal of Molecular Sciences

MDPI

Supplementary Table S5 Key salt-responsive genes corresponding to each module (* mark as curated gene with publication in RAP-DB)

No.	Locus MSU/ RAP ID	Gene symbol	Module	Centrality	Gene product name	Reported features and function	References
1	LOC_Os12g08020 Os12g0180800	OsbHLH179, bHLH179	Grey	DG, CN, BW, CC	Similar to OSIGBa0158F05.9 protein	The basic helix-loop-helix (bHLH) transcription factors play important roles in diverse cellular and molecular processes. GO enrichment analysis for the modules showed that some TabHLHs (wheat, *Triticum aestivum*) were involved in the control of several biological processes, such as tapetal PCD, lipid metabolism, iron absorption, stress responses and signal regulation	[1]
2	LOC_Os07g42885 Os07g0621201		Grey	DG, CN, CC	retrotransposon protein, putative, Ty1-copia subclass, expressed		
3	LOC_Os08g01670 Os08g0108100	PMEI28, OsPMEI28	Grey	DG, CN, CC	invertase/pectin methylesterase inhibitor family protein, putative, expressed	Pectin, one of the major cell wall polysaccharides. Quantification of cell wall-bound methylesters indicated that the degree of pectin methylesterification is developmentally regulated; in particular, higher PMEI (pectin methylesterase inhibitors) activities were detected in cell wall proteins prepared from young leaves. Subcellular localization indicated that OsPMEI8 is targeted to the middle lamella and OsPMEI12 is localized in the plasma membrane and nucleus.	[2]

4	LOC_Os07g35720 Os07g0541600		Grey	DG, CN, BW, CC	Similar to cDNA clone:002-112-D06, full insert sequence		
5	LOC_Os08g20480 Os08g0299700	OsFbox435, OsF0317, OsFBO20, FBO20	Grey	DG, CN, CC	OsFBO20 - F-box and other domain containing protein, expressed, F-box-type E3 ubiquitin ligase O20	FBX (F-Box) proteins comprise the target recognition subunit of SCF-type ubiquitin-protein ligases, where they individually recruit specific substrates for ubiquitylation. It appears that the FBX superfamily has independently undergone substantial birth/death in many plant lineages, with its size and rapid evolution potentially reflecting a central role for ubiquitylation in driving plant fitness. The ubiquitylation modification plays a crucial role in various cellular processes, including protein degradation, signal transduction, and the regulation of gene expression.	[3, 4]

6	LOC_Os12g08000 Os12g0180600		Grey	DG, CN, BW, CC	5-nucleotidase domain-containing protein, putative, expressed		
7	LOC_Os01g05120 Os01g0144500		Grey	DG, CN, CC	Ubiquitin fusion degradation protein UFD1 domain containing protein		

8	LOC_Os07g43540<br>Os07g0628600	ORC6,<br>OsORC6	Grey	DG, CN,<br>BW, CC	Origin recognition complex subunit 6 (ORC6) family protein, expressed	1.) Core DNA replication genes in rice. Core DNA replication proteins mediate the initiation, elongation, and Okazaki fragment maturation functions of DNA replication. The core DNA replication machinery is highly conserved across plant species and displays many features in common with other eukaryotes and some characteristics that are unique to plants<br>2.) OsORC6 (Os07g0628600) was down-regulated by Zn deficiency in both roots and shoots	[5, 6]
9	LOC_Os11g09700<br>Os11g0202500		Grey	DG, CN,<br>BW, CC	anthocyanidin 5,3-O-glucosyltransferase, putative, expressed, UDP-glucuronosyl/ UDP-glucosyltransferase domain containing protein	1.) stabilization of pigments, enhancement of solubility, storage of secondary metabolites, regulation of plant growth regulators<br>2.) exposure to atrazine can trigger specific GT (glycosyltransferase) genes and enzyme activities in rice<br>2.) related to dwarf and early-senescence leaf1 *(del1)* gene<br>3.) UDP-glucose-dependent glycosyltransferases (UGTs) were consistently induced in response to biotic and abiotic stress	[7-10]

10	LOC_Os07g02120 Os07g0111900		Grey	DG, CN	flavin-containing monooxygenase family protein, putative, expressed	Ascorbic acid (AsA), or vitamin C, is a strong antioxidant that prevents oxidative damage to cellular components and plays an essential role in plant defense response. GDP-D-mannose pyrophosphorylase (GMP or VTC1) is an enzyme that generates GDP-D-mannose for AsA, cell wall, and glycoprotein synthesis. Os07g0111900 is top 20 down-regulated differentially expressed genes (DEGs) in OsVTC1-1 RI1-2 line at 24 h after inoculation with rice blast 10100 isolate.	[11]
11	LOC_Os07g02192 Os07g0112600		Grey	DG, CN	expressed protein		
12	LOC_Os07g43560 Os07g0628700		Grey	DG, CN, BW, CC	TKL_IRAK_DUF26-lc.24 - DUF26 kinases have homology to DUF26 containing loci, expressed	1.) causative genes in the salt-tolerant response by GWAS<br><br>2.) LOC_Os07g43560 downregulated under pre-anthesis drought stress in rice	[12, 13]
13	LOC_Os07g43570 Os07g0628900		Grey	DG, CN, BW, CC	TKL_IRAK_DUF26-lc.25 - DUF26 kinases have homology to DUF26 containing loci, expressed	LOC_Os07g43570, a gene related to signaling transduction, up-regulated under drought conditions	[14]
14	LOC_Os07g02110 Os07g0111800		Grey	DG, CC	Conserved hypothetical protein		
15	LOC_Os11g11130 Os11g0218200		Grey	DG, CN, BW, CC	Similar to RNApol24, expressed protein		

16	LOC_Os12g07990<br>Os12g0180500	RLCK361,<br>OsUSP40	Grey	DG, CN, BW, CC	protein kinase family protein, putative, expressed	1.) Receptor-like cytoplasmic kinases (RLCKs) in plants belong to the super family of receptor-like kinases (RLKs). Majority of the stress-responsive OsRLCKs were also found to be localized within mapped regions of abiotic stress QTLs. OsRLCK361 (LOC_Os12g07990)<br><br>2.) Universal stress proteins (USPs) have received much attention in the field of plant science due to their stress-specific transcriptional regulation. Strong evidence for the utility of OsUSP genes in building multi-stress tolerant plants. Most OsUSP genes exhibited moderate to high expression levels in all tissues exception of OsUSP40 (Os12g0180500)	[15, 16]
17	LOC_Os01g02960<br>Os01g0119500			DG, CN	Protein of unknown function DUF1618 domain containing protein	Candidate gene located in four QTLs regions involve cold tolerance in rice	[17]
18	LOC_Os02g57290<br>Os02g0817900	CYP97A4,<br>OsCYP97A4	Grey	DG, CN, BW, CC	cytochrome P450, putative, expressed	Rice Cyt P450 gene CYP97A4 encoding a carotenoid b-ring hydroxylase was shown to be involved in lutein biosynthesis, preferentially expressed in leaf and encoded-protein localized at the subcellular level to the chloroplasts	[18]
19	LOC_Os04g10150<br>Os04g0180300		Grey	DG, CN, CC	Similar to OSIGBa0105N24.3 protein	Candidate gene in QTL of brown rice (whole grain with bran) ionome (sum total of such mineral composition)	[19]

20	LOC_Os08g03002 Os08g0124100	OsLecRK IX.1	Grey	DG, CN, CC	Concanavalin A-like lectin/glucanase, subgroup domain containing protein	OsLecRK IX.1 (Os08g0124100) is PTI-associated genes (pattern-triggered immunity). Overexpressing SDG703 (SET domain group 703) revealed the downregulation of PTI. SDG703 suppresses the expression of defense-related genes through the promotion of histone methylation	[20]
21	LOC_Os01g05530 Os01g0148400		Grey	DG, CN, CC	Brix domain containing protein	CSA (carbon starved anther) clearly plays a role in directing sugar metabolism in anthers, it may also have other regulatory effects on plant growth and development. CSA has some common functions in the anther and leaf. These genes encode a brix domain containing protein (LOC_Os01g05530), a protein involved in vacuolar import.	[21]
22	LOC_Os01g05510 Os01g0148100		Grey	DG, CN, CC	Conserved hypothetical protein		
23	LOC_Os01g05610 Os01g0149400	H2B.3, OsH2B.3	Grey	CN, CC	Core histone H2A/H2B/H3/H4 domain containing protein, putative, expressed	Rice OsUBR7 (plant height-related gene) modulates plant height by regulating histone H2B monoubiquitination and cell proliferation. Knockout of OsUBR7 caused fewer cells in internodes, resulting in a semi-dwarf phenotype	[22]

24	LOC_Os01g05400 Os01g0147001		Grey	DG, CN, CC	Similar to H0212B02.9 protein	two nsSNPs (non-synonymous SNP) were found in Os01g0147001 that encodes for glycosyltransferase in the study of genomewide SNP analysis from the genome resequencing of four Malaysian pigmented rice varieties, representing two black and two red rice varieties.	[23]
25	LOC_Os02g57310 Os02g0818500*	Pib	Grey	DG, CN, CC	Blast disease resistance (R) protein, pib, putative, expressed	Plants usually keep resistance (R) proteins in a static state under normal conditions to avoid autoimmunity and save energy for growth, but R proteins can be rapidly activated upon perceiving pathogen invasion. Pib, the first cloned blast disease R gene in rice, encoding a nucleotide-binding leucine-rich repeat (NLR) protein, mediates resistance to the blast fungal (*Magnaporthe oryzae*) isolates carrying the avirulence gene AvrPib.	[24]
26	LOC_Os01g05680 Os01g0150100*	PGGTI-BETA, OsPGGT I-beta, PGGT I-beta, OsPGGT I-β	Grey	DG, CN, CC	geranylgeranyl transferase type-1 subunit beta, putative, expressed	Protein prenylation plays an important role in rice development. Protein-protein interactions showed that the farnesyl-transferase(OsPFT)/geranylgeranyltransferae-I (OsPGGT I-α) protein interation with together with OsPFT-β and OsPGGT I-β (LOC_Os01g05680)	[25]
27	LOC_Os01g05520 Os01g0148200		Grey	CN, BW, CC	Hypothetical protein		

28	LOC_Os08g09820 Os08g0198200		Grey	DG, CN, BW, CC	peptidase aspartic family protein, putative, expressed		
1	LOC_Os11g15520 Os11g0261600	SUB62, OsSub62	Green	DG, CN, CC	OsSub62 - Putative Subtilisin homologue, expressed	Subtilisin-like proteases (SUBs), which are extensively distributed in three life domains, affect all aspects of the plant life cycle, from embryogenesis and organogenesis to senescence. SUBs associated with rice caryopsis (one-seeded fruit where the seed coat is tightly fused with the ovary wall) development.	[26]
2	LOC_Os11g15370 Os11g0260200		Green	DG, CN, CC	sulfotransferase domain containing protein, expressed	Os11g0260200 was in the list of potential candidate genes in QTLs associated to total dry weight (TDW) with tolerance to low soil fertility.	
3	LOC_Os08g31910 Os08g0413500		Green	DG, CN, CC	expressed protein		
4	LOC_Os06g12910 Os06g0235800		Green	DG, CN, BW, CC	XPA-binding protein 2, putative, expressed		
5	LOC_Os06g13040 Os06g0237400		Green	DG, CN, BW, CC	glycosyl hydrolases family 16, putative, expressed, Similar to xyloglucan endotransglucosylase/hydrolase protein 1		
6	LOC_Os06g12470 Os06g0230400		Green	DG, CN, CC	retrotransposon protein, putative, Ty3-gypsy subclass, expressed		

7	LOC_Os08g43000 Os08g0543050		Green	DG, CN, CC	CC-NBS-LRR, putative, expressed	Regions of amino acid conservation in the NBS domain of NBS-LRR resistance proteins facilitated the PCR isolation of eight resistance gene analog (RGA) sequences from genomic DNA of rice, barley, and *Aegilops tauschii*. The positions of orthologous RGAs are conserved between barley and wheat. RGA loci were identified in the vicinity of barley leaf rust resistance loci	[27]
8	LOC_Os06g24180 Os06g0349700*	OsF5HL, F5HL, CYP84A7	Green	DG, CN	Ferulate-5-hydroxylase	Ferulate-5-hydroxylase (FSH) is a cytochrome P450-dependent monooxygenase that catalyses the hydroxylation of ferulic acid, coniferaldehyde, and coniferyl alcohol in the pathways leading to sinapic acid and the syringyl unit of lignin. OsFSHL is highest in young leaves, whereas that of OsFSHL2 is greatest in mature leaves. In the roots and stems, transcription levels for both genes are markedly low, suggesting OsFSHL and OsFSHL2 genes belong to the CYP84 subfamily and that their expressions are tissue specific	[28]
9	LOC_Os02g43490 Os02g0651200		Green	DG, CN, BW, CC	Conserved hypothetical protein		
10	LOC_Os07g29810 Os07g0481300		Green	DG, CN, CC	disease resistant protein, identical, putative, expressed		

11	LOC_Os07g10950 Os07g0210800	MSL28, OsMSL28	Green	DG, CN, CC	expressed protein	Trihelix transcription factors play a role in plant growth, development, and various stress responses. These OsMSLs (Myb/SANT-LIKE) were located on twelve chromosomes.<br>The OsMSLs responded to abiotic stresses including drought and high salt stress and stress signal molecule including ABA (abscisic acid), hydrogen peroxide. Os07g0210800 is trihelix genes family in rice	[29]
12	LOC_Os06g33350 Os06g0524700		Green	DG, CN, CC	plant protein of unknown function domain containing protein, expressed		
13	LOC_Os11g15590 Os11g0262200		Green	DG, CN, CC	Hypothetical conserved gene		
14	LOC_Os08g30634 Os08g0396700		Green	DG, CN, CC	NB-ARC domain containing protein		
15	LOC_Os08g32160 Os08g0417000		Green	DG, CN, CC	2OG-Fe(II) oxygenase domain containing protein		
16	LOC_Os08g31970 Os08g0414600		Green	DG, CN, CC	Six-bladed beta-propeller		
17	LOC_Os08g30719 Os08g0397800		Green	DG, CN, CC	expressed protein		

18	LOC_Os08g31219 Os08g0404200*	S27, RPL27-2, mtRPL27a, S27(t), S27(t)*, rpl27-2, rpl27	Green	DG, CN, CC	Mitochondrial ribosomal protein L27	1.) plant mitochondrial rpl27 (mitochondrial ribosomal protein) have been transferred previously from the mitochondrial genome to the nuclear genome. The promoter shuffling resulted from a recent segmental duplication through inter- and intrachromosomal recombination events.<br><br>2.) The reciprocal loss of duplicated genes encoding mitochondrial ribosomal protein L27 causes hybrid pollen sterility in F1 hybrids of the cultivated rice *Oryza sativa* and its wild relative *O. glumaepatula*. Functional analysis revealed that this gene is essential for the later stage of pollen development.	[30, 31]
19	LOC_Os08g32690 Os08g0422600*	SAD1	Green	DG, CN, CC	RNA polymerase I subunit A34.5	The DWARF14 (D14) gene of rice functions within the signaling pathway of strigolactones, a group of plant hormones that inhibits shoot branching. sad1- 1 (super apical dormant) mutant from a suppressor screen of d14-1 showed suppress growth of tillers. SAD1 encodes an ortholog of RPA34.5, a subunit of RNA polymerase I (Pol I). The proper ribosome function is a prerequisite for normal development in plants.	[32]
20	LOC_Os08g31880 Os08g0413100		Green	DG, CN, CC	Protein of unknown function DUF1262 domain containing protein		

21	LOC_Os08g32630 Os08g0422000		Green	DG, CN, CC	FAD dependent oxidoreductase, putative, expressed, Similar to CM0545.530.nc protein (Fragment)	Co-expressors of rice NDC1 (LOC_Os06g11140) and MENG. Arabidopsis NAD(P)H DEHYDROGENASE C1 (NDC1; At5g08740), which encodes a member of the type II NAD(P)H dehydrogenase family, also blocks the methylation the naphthalenoid ring. demethylnaphthoquinone methyltransferase (MENG; At1g23360; EC 2.1.1.163), which catalyzes the last step of phylloquinone biosynthesis in plastids.	[33, 34]
22	LOC_Os07g29770 Os07g0481000		Green	DG, CN, CC	zinc finger protein, putative, expressed, Similar to Pollen-specific kinase partner protein	Genes differentially expressed in OsAS84, a root system architecture (RSA). OsAS83, lowland and OsAS84, upland	[35]
23	LOC_Os06g13050 Os06g0237502*	YPD1, OsYPD1	Green	DG, CN, CC	peroxidase family protein, expressed	The novel rice LRR-like1 protein YPD1 (yellow and premature dwarf 1) affects chloroplast development and leaf senescence	[36]
24	LOC_Os06g13210 Os06g0239500		Green	DG, CN, CC	peptide transporter PTR2, putative, expressed, TGF-beta receptor, type I/II extracellular region family protein	transport of di-/tripeptides, amino acids, nitrate uptake and transport, root development under salt stress condition	[37, 38]
25	LOC_Os08g30590 Os08g0396500		Green	DG, CN, CC	C1-like domain containing protein, expressed		

1	LOC_Os02g38780 Os02g0599700	PP2C19, OsPP2C19, OsPP29	Turquoise	DG, CN, CC	Protein phosphatase 2C, manganese/magnesium aspartate binding site domain containing protein	LOC_Os02g38780 (OsPP29) is PP2C class of serine/threonine phosphatases. OsPPs present in tandem duplication in rice genome. Expression profiling and analysis indicate the involvement of this large gene family in a number of signaling pathways triggered by abiotic stresses and their possible role in plant development.	[39]
2	LOC_Os03g44180 Os03g0643800		Turquoise	DG, CC	hydroquinone glucosyltransferase, putative, expressed	Transposons or transposable elements (TEs)-derived accessible chromatin regions (ACRs) play essential roles in multiple biological processes by interacting with trans-acting factors. TE-derived tissue-specific ACRs were located at the upstream of subsets of functional tissue-specific genes. For example, a MULE (Mutator-like elements)-derived ACR was located in the promoter of a hydroquinone glucosyltransferase encoding gene (LOC_Os03g44180), preferentially expressed in root (RT). ACRs are likely involved in the regulation of tissue development, rice domestication and functional divergence of duplicated genes.	[40]

3	LOC_Os08g02800 Os08g0121400		Turquoise	DG, CN, CC	ribosome, putative, expressed	LOC_Os08g02800 a DEG in the experiment of floret-opening time (FOT). The *japonica* rice cultivar G23 with early FOT was crossed with regular *japonica* rice Nangeng 9108 (NG9108). FOT refers to the time between florets opening and closing within a day, and is a crucial agricultural trait for reproductive development, thermal tolerance and hybrid breeding in rice.	[41]
4	LOC_Os10g22170 Os10g0366400		Turquoise	DG, CN, CC	Conserved hypothetical protein	Panicle architecture is an important component of agronomic trait in rice, which is also a key ingredient that could influence yield and quality of rice. Os10g0366400 main up-expressed in transcriptional profile of rice panicles (NIL-GW8 and NIL-gw8^{Amol}) at three different stages of panicle development	[42]
5	LOC_Os10g24100 Os10g0382600		Turquoise	DG, CN, CC	Conserved hypothetical protein	The nitrogen use efficiency (NUE) of plants is defined as their efficiency at utilizing N from the soil. NUE has two major component traits, namely N uptake and N utilization. Os10g0382600 was a candidate gene that had unknown functions of genome-wide transcriptome analysis of two rice genotypes, IR 64 (IR64) and Nagina 22 (N22) under optimal (N^+) and chronic starvation (N^-) of nitrogen (N) from 15-day-old root and shoot tissues.	[43]

6	LOC_Os11g07100<br>Os11g0172133		Turquoise	DG, CN, CC	receptor-like protein kinase precursor, putative, expressed	List of genes located in the QTL in chromosome 11 detected by QTL-seq analysis in the study of bacterial panicle blight (BPB) and sheath blight (SB) using linkage mapping combined with a bulk segregant analysis with whole-genome sequence data (QTL-seq)	[44]
7	LOC_Os04g08070<br>Os04g0162600		Turquoise	DG, CN, CC	Protein of unknown function DUF295 family protein	Os04g0162600 synteny between *Brachypodium* (a genus of plants in the grass family) and rice genes in the S-locus (single locus) region of self-incompatibility (prevents self-fertilization).	[45]
8	LOC_Os02g28130<br>Os02g0483000		Turquoise	DG, CN, CC	fasciclin-like arabinogalactan protein 8 precursor, putative, expressed		
9	LOC_Os01g39840<br>Os01g0580300		Turquoise	DG, CN, CC	Conserved hypothetical protein	Os01g0580300, candidate genes differentially expressed for galactose-related genes in the study of grain number per panicle (GNPP) in rice	[46]
10	LOC_Os02g06140<br>Os02g0155900		Turquoise	DG, CN, CC	retrotransposon protein, putative, unclassified, expressed	LOC_os02g06140 that were upregulated >2-fold may play an important role in its immunity against *Xoo (Xanthomonas oryzae pv. oryzae)* infection.	[47]
11	LOC_Os02g38890<br>Os02g0601000		Turquoise	DG, CN, CC	Conserved hypothetical protein	LOC_Os02g38890 was designed as non-synonymous SNP marker for bacterial panicle blight (BPB) and sheath blight (SB)	[48]

12	LOC_Os11g07710 Os11g0179400	DIR38, OsDIR38, OsjDIR38	Turquoise	DG, CN, CC	dirigent, putative, expressed	Dirigent (DIR) proteins members have been shown to play essential roles in plant growth, development and adaptation to environmental changes. Analysis of the RNA sequencing data indicates that OsjDIR genes respond to a wide range of environmental factors, and most OsjDIR genes have a high expression level in roots.	[49]
13	LOC_Os02g42100 Os02g0631801		Turquoise	DG, CN, BW, CC	Hypothetical conserved gene		
14	LOC_Os08g02840 Os08g0121800		Turquoise	DG, CN, CC	Ribosomal protein S21 family protein		
15	LOC_Os01g38650 Os01g0567200		Turquoise	CN, BW, CC	Protein of unknown function DUF3778 domain containing protein	Plant transcription factors (TFs), such as basic helix-loop-helix (bHLH) and AT-rich zinc-binding proteins (PLATZ), play critical roles in regulating the expression of developmental genes in cereals.<br><br>Os01g0567200 showed expression patterns similar to TaPGS1 overexpressed. bHLH protein TaPGS1 (*T. aestivum* Positive Regulator of Grain Size 1) specifically expressed in the seeds at 5–20 days post-anthesis in wheat	[50]
16	LOC_Os11g08970 Os11g0195000		Turquoise	DG, CN, BW, CC	heat shock factor, putative, expressed		

17	LOC_Os11g08480 Os11g0188100		Turquoise	DG, CN, BW, CC	Similar to inositol or phosphatidylinositol kinase/ phosphotransferase, alcohol group as acceptor		
18	LOC_Os01g24080 Os01g0343400		Turquoise	DG, CN, BW, CC	Conserved hypothetical protein	List of annotated genes within the LD blocks of significant SNPs, candidate genes associated with root growth angle	[51]
19	LOC_Os02g06170 Os02g0156100		Turquoise	DG, CN, BW, CC	Conserved hypothetical protein		
20	LOC_Os02g38860 Os02g0600650		Turquoise	DG, CN, BW, CC	Conserved hypothetical protein		
21	LOC_Os11g43895 Os11g0660400	OsWD40-191	Turquoise	DG, CN, BW, CC	expressed protein	OsWD40 genes may perform their diverse functions by complex network, thus were predictive for understanding their biological pathways. The analysis also revealed that OsWD40 genes might interact with each other to take part in metabolic pathways, suggesting a more complex feedback network.	[52]
22	LOC_Os11g10790 Os11g0214100	DIR42, OsDIR42, OsjDIR42	Turquoise	DG, CN, BW, CC	Plant disease resistance response protein domain containing protein	Dirigent (DIR) proteins members have been shown to play essential roles in plant growth, development and adaptation to environmental changes. Analysis of the RNA sequencing data indicates that OsjDIR genes respond to a wide range of environmental factors, and most OsjDIR genes have a high expression level in roots.	[49]

23	LOC_Os02g15720<br>Os02g0257001		Turquoise	DG, CN, BW, CC	retrotransposon protein, putative, Ty3-gypsy subclass, expressed		
1	LOC_Os01g51410<br>Os01g0711400		Yellow	DG, CN, BW, CC	glycine dehydrogenase, putative, expressed, Similar to Victorin binding protein	A differentially expressed proteins list changes greater than 1.5-fold after the first 24 h of cold treatment	[53]
2	LOC_Os01g08700<br>Os01g0182600*	GI, OsGI, Gi, Os-GI	Yellow	DG, CN, BW, CC	Orthologue of the Arabidopsis GIGANTEA	1.) OsGI involve the photoperiodic regulation of flowering<br>2.) GI gene in transgenic rice, caused late flowering under both short-day and long-day condition<br>3.) key flowering time regulator, OsGIGANTEA (OsGI) might be a negative regulator in the osmotic stress response in rice. Mutation of OsGI conferred tolerance to osmotic stress generated by polyethylene glycol (PEG), increased proline and sucrose contents, and accelerated stomata movement	[54-56]

3	LOC_Os03g20700<br>Os03g0323200	CHLH,<br>OsChlH,<br>ChlH,<br>OsCHLH	Yellow	DG, CN,<br>CC	Magnesium chelatase subunit ChlH	1.) Mesophyll cells in the OsCHLH rice mutant derive ATP from mitochondrial respiration, and this is critical for the normal function of plasma membrane outward-rectifying K^+ channels. The OsCHLH mutant which lacks chlorophyll in the thylakoids is unable to fix CO_2 and exhibits reduced growth<br><br>2.) Magnesium-protoporphyrin IX monomethyl ester cyclase (MPEC) catalyzes the conversion of MPME to divinyl protochlorophyllide (DVpchlide). This is an essential enzyme during chlorophyll (Chl) biosynthesis and YL-1 (yellow-leaf 1) encodes a subunit of MPEC. The gene upstream of Chl biosynthesis Mg chelatase H subunit (CHLH) highly expressed in yl-1 mutant	[57, 58]
4	LOC_Os07g37240<br>Os07g0558400	CP29, OsCP29,<br>LHCB4, Lhcb4,<br>OsLHCB4	Yellow	DG, CN,<br>CC	chlorophyll A-B binding protein, putative, expressed	1.) Os07g0558400 (CP29) is genes encoding light-harvesting proteins<br><br>2.) OsLhcb4 (Os07g0558400) senescence down-regulated genes (SDGs), decreased much faster in osphyB-2 (Rice Phytochrome B) mutants. OsPhyB negatively regulates dark- and starvation-induced leaf senescence	[59, 60]

5	LOC_Os03g03910 Os03g0131200	NOE1, CATC, OsCat, OsCatC, OsCATC, noe1, catC, OsNOE1, CAT3, OsCAT3, LLM9428, OsLLM9428	Yellow	CN, CC	catalase domain containing protein, expressed	Nitric oxide (NO) is a key redox-active, small molecule involved in various aspects of plant growth and development. NOE1 (nitric oxide excess1) encoded a rice catalase, OsCATC. Both NO and SNOs (S-nitrosothiol) are important mediators in the process of H_2O_2-induced leaf cell death in rice	[61]
6	LOC_Os02g10390 Os02g0197600	LHCA3, OsLhca3, Lhca3	Yellow	CN, BW, CC	Chlorophyll a/b-binding protein type III (Fragment)	Os02g0197600 was a candidate gene around main-effect QTNs of rice grain length in main crop and found to be related to cytokinin	[62]
7	LOC_Os06g45820 Os06g0669400*	FTSH2, OsFtsH2, FtsH2	Yellow	DG, CN, CC	ATP-dependent zinc metalloprotease	Filamentation temperature-sensitive H (FtsH) is an ATP-dependent zinc metalloprotease with ATPase activity, proteolysis activity and molecular chaperone-like activity. osftsh2 mutants could not form normal chloroplasts and had lost photosynthetic autotrophic capacity. OsFtsH2 could be essential for chloroplast development in rice	[63]

8	LOC_Os07g34589 Os07g0529800*	Os-eIF-1, eIF-1, OseIF1, GOS2, OsGOS2, SUI1, PtfS	Yellow	DG, CN, CC	translation initiation factor SUI1, putative, expressed	1.) The eIF-1 (eukaryotic translation initiation factor) over-expressing rice showed improved growth under salt stress that was correlated with maintenance of photosynthetic activity and reduced Na^+ and Cl^- accumulation in leaves<br><br>2.) Expression of OseIF1 was increased by salt stress and upregulated after exogenous ABA and mannitol treatments, suggesting that its induction is related to the water-deficit effect of high salt	[64, 65]
9	LOC_Os07g04840 Os07g0141400	PSBP, PsbP, OsPsbP, psbP	Yellow	DG, CN, CC	Similar to 23 kDa polypeptide of photosystem II, PsbP, putative, expressed	Os07g0141400 was annotated as a 23 kDa polypeptide of photosystem II (PSII) and is also known as PsbP or OsPsbP. OsPsbP seems to regulate leaf color in rice	[66]
10	LOC_Os01g73940 Os01g0971000		Yellow	DG, CN, CC	expressed protein		
11	LOC_Os01g10400 Os01g0200700*	MTI3A, OsMT-3a, OsMT3a, MT3a, OsMT-I-3a, MT-I-3a, MTe, met2, OsMT3, MT3, OsMTI-3a, MTI-3a	Yellow	DG, CN, CC	Metallothionein-like protein	Metallothioneins (MT) are primarily involved in metal chelation. The rice metallothionein-like gene *OsMT-3a* transgenic plants showed substantially increased salinity tolerance (NaCl), drought tolerance (PEG), and heavy metal tolerance (CdCl2) as individual stresses, as well as different combinations of these stresses.	[67]

12	LOC_Os06g21590 Os06g0320500	LHCA1, OsLhca1, Lhca1	Yellow	DG, CN, BW, CC	chlorophyll A-B binding protein, putative, expressed, Similar to Light-harvesting complex I (Fragment)	1.) The light-harvesting complex (LHC) of PSI (LHCI) subunits, Lhca1 dissociation under in Iron (Fe) deficiency may be due to increased levels of reactive oxygen species.<br>2.) OsWHY1 contributes to early chloroplast development and normal seedling survival in rice. AtWHY1 is involved in light adaptation by interacting with light-harvesting protein complex I (LHCA1) and affecting the expression of genes related to photosystem I (PS I) and light-harvesting complexes (LHCIs)	[68, 69]
13	LOC_Os05g48630 Os05g0560000	PSAH, PsaH, GOS5, OsPSAH, OsGOS5	Yellow	DG, CN, CC	Photosystem I reaction center subunit VI, chloroplast precursor (PSI-H) (Light-harvesting complex I 11 kDa protein) (GOS5 protein)	Photosynthetic parameters, including net photosynthetic rate, transpiration rate, stomatal conductance, intercellular CO_2 concentration, chlorophyll concentration and the chlorophyll fluorescence, were decreased in rice exposed to high-Zn treatment. The expression levels of Os05g48630 (PsaH) decreased in Si-deprived plants under high-Zn stress	[70]

14	LOC_Os02g47020 Os02g0698000	OsPrk, Prk	Yellow	DG, CN, CC	Similar to Phosphoribulokinase, chloroplast precursor (EC 2.7.1.19) (Phosphopentokinase) (PRKase) (PRK). phosphoribulokinase/Uridine kinase family protein, expressed	Phosphoribulokinase (PRKase) plays an important role in regulating the flow of sugar through the Calvin cycle. OsPrk expression was down-regulated by externally applied NaCl, ABA, MeJA(methyl jasmonate) and glucose over 24 hr, whereas it was up-regulated by GA after 24 hr treatments. These results also indicated that OsPrk gene expression is modulated by these factors at multiple levels	[71]
15	LOC_Os01g45274 Os01g0639900*	BETACA1, OsβCA1, OsbCA1, OsCA, CA, OsbetaCA1, betaCA1, CA1	Yellow	DG, CN, BW, CC	carbonic anhydrase, chloroplast precursor, putative, expressed, Beta-carbonic anhydrase (EC:4.2.1.1)	A chloroplast-located gene coding for b-carbonic anhydrase (bCA) was found to be involved in carbon assimilation and the CO_2-mediated stomatal pore response in rice. The absence of OsbCA1 reduces photosynthetic efficiency, triggers the opening of stomatal pores and finally decreases their sensitivity to CO_2 fluctuation	[72]
16	LOC_Os04g38600 Os04g0459500	GAPDH	Yellow	DG, CN, CN	glyceraldehyde-3-phosphate dehydrogenase, putative, expressed	LOC_Os04g38600 (encoding a glyceraldehyde-3- phosphate dehydrogenase), a major hub gene detects by co-expression network analysis, suggesting that photosynthetic adaptation via NADP(H) homeostasis contributes to drought tolerance in rice	[73]

1	LOC_Os06g19800 Os06g0302000		Red	DG, CN, CC	BURP domain containing protein, expressed	A down regulated differentially expressed genes (DEGs) list in the roots of OsMYB305-OE lines. OsMYB305 overexpression suppressed cellulose biosynthesis under low-nitrogen condition, thereby freeing up carbohydrate for nitrate uptake and assimilation and enhancing rice growth	[74]
2	LOC_Os06g37410 Os06g0570900	OsbHLH030	Red	DG, CN, CC	helix-loop-helix DNA-binding domain containing protein, expressed	There are167 bHLH genes in rice genome. OsbHLH107 and its homologs are important regulators of grain size development	[75, 76]
3	LOC_Os01g39680 Os01g0578700*	SAM, SaM, SaM-, SaM+	Red	DG, CN, CC	Small ubiquitin-like modifier (SUMO) E3 ligase-like protein	The complex locus Sa comprises two adjacently located genes, SaF and SaM, which interact to cause abortion of pollen grains carrying the japonica allele in japonica-indica hybrids.	[77]
4	LOC_Os06g19620 Os06g0300300		Red	DG, CN, CC	SFT2, putative, expressed		
5	LOC_Os06g19430 Os06g0298100		Red	DG, CN, CC	Conserved hypothetical protein		
6	LOC_Os01g39670 Os01g0578500*	SAF, SaF, OsFbox019, OsFbox19, Os_F0070, OsFBD1, FBD1	Red	DG, CN, BW, CC	OsFBD1 - F-box and FBD domain containing protein, expressed	Hybrid male sterility, Sa, comprises two adjacent genes, SaM and SaF, encoding a small ubiquitin-like modifier E3 ligase-like protein and an F-box protein. A two-gene/three component interaction model for hybrid male sterility system	[78, 79]
7	LOC_Os06g17700 Os06g0286146		Red	DG, CN, CC	embryogenesis transmembrane protein, putative, expressed		

8	LOC_Os06g35870<br>Os06g0552000		Red	DG, CN, CC	lectin protein kinase family protein, putative, expressed,		
9	LOC_Os02g30960<br>Os02g0513700		Red	DG, CN, CC	transposon protein, putative, Ac/Ds sub-class, expressed		
10	LOC_Os06g19610<br>Os06g0300200		Red	DG, CN, CC	oxidoreductase, short chain dehydrogenase/reductase family, putative, expressed		
1	LOC_Os12g10570<br>Os12g0207500	ATPASE 3, ATPase3	Blue	DG, CN, BW, CC	ATP synthase subunit beta, putative, expressed	1.) Differentially expressed genes involved in carbon and nitrogen metabolism<br>2.) salt stress increased the expression levels ATPase3	[80, 81]
2	LOC_Os10g21268<br>Os10g0356000	OsrbcL1, rbcL1	Blue	DG, CN, BW, CC	ribulose bisphosphate carboxylase large chain precursor, putative, expressed	OsrbcL1, a photosynthesis-related proteins. OsGIRP1 mediates proteolysis of 2 substrates, OsrbcS1 and OsrbcL1, via the 26S proteasome degradation pathway. OsGIRP1 acts as a negative regulator of GR response to mediate the degradation of photosynthesis-related proteins	[82]
3	LOC_Os10g21264<br>Os10g0355800	AtpB	Blue	DG, CN, BW, CC	ATP synthase epsilon chain, putative, expressed	candidate genes identified by GWAS exposed to short term saline conditions (50 mM NaCl)	[83]
	Os01g0791033						
1	-<br>Os12g0525300		Brown	DG, CN, CC	Hypothetical protein		
2	-<br>Os12g0524201		Brown	DG, CN, CC	Hypothetical conserved gene		

References

1. Wei, K.; Chen, H., Comparative functional genomics analysis of bHLH gene family in rice, maize and wheat. *BMC plant biology* **2018,** 18, 1-21.
2. Nguyen, H. P.; Jeong, H. Y.; Kim, H.; Kim, Y. C.; Lee, C., Molecular and biochemical characterization of rice pectin methylesterase inhibitors (OsPMEIs). *Plant Physiology and Biochemistry* **2016,** 101, 105-112.
3. Hua, Z.; Zou, C.; Shiu, S.-H.; Vierstra, R. D., Phylogenetic comparison of F-Box (FBX) gene superfamily within the plant kingdom reveals divergent evolutionary histories indicative of genomic drift. *PLoS One* **2011,** 6, (1), e16219.
4. Wang, S.; Lv, X.; Zhang, J.; Chen, D.; Chen, S.; Fan, G.; Ma, C.; Wang, Y., Roles of E3 ubiquitin ligases in plant responses to abiotic stresses. *International Journal of Molecular Sciences* **2022,** 23, (4), 2308.
5. Shultz, R. W.; Tatineni, V. M.; Hanley-Bowdoin, L.; Thompson, W. F., Genome-wide analysis of the core DNA replication machinery in the higher plants Arabidopsis and rice. *Plant Physiology* **2007,** 144, (4), 1697-1714.
6. Zeng, H.; Zhang, X.; Ding, M.; Zhu, Y., Integrated analyses of miRNAome and transcriptome reveal zinc deficiency responses in rice seedlings. *BMC Plant Biology* **2019,** 19, (1), 1-18.
7. Ko, J. H.; Kim, B. G.; Hur, H.-G.; Lim, Y.; Ahn, J.-H., Molecular cloning, expression and characterization of a glycosyltransferase from rice. *Plant cell reports* **2006,** 25, 741-746.
8. Leng, Y.; Yang, Y.; Ren, D.; Huang, L.; Dai, L.; Wang, Y.; Chen, L.; Tu, Z.; Gao, Y.; Li, X., A rice PECTATE LYASE-LIKE gene is required for plant growth and leaf senescence. *Plant Physiology* **2017,** 174, (2), 1151-1166.
9. Brazier-Hicks, M.; Gershater, M.; Dixon, D.; Edwards, R., Substrate specificity and safener inducibility of the plant UDP-glucose-dependent family 1 glycosyltransferase super-family. *Plant biotechnology journal* **2018,** 16, (1), 337-348.
10. Lu, Y. C.; Yang, S. N.; Zhang, J. J.; Zhang, J. J.; Tan, L. R.; Yang, H., A collection of glycosyltransferases from rice (Oryza sativa) exposed to atrazine. *Gene* **2013,** 531, (2), 243-252.
11. Lamanchai, K.; Smirnoff, N.; Salmon, D. L.; Ngernmuen, A.; Roytrakul, S.; Leetanasaksakul, K.; Kittisenachai, S.; Jantasuriyarat, C., OsVTC1-1 Gene Silencing Promotes a Defense Response in Rice and Enhances Resistance to Magnaporthe oryzae. *Plants* **2022,** 11, (17), 2189.
12. Ahmad, H.; Zafar, S. A.; Naeem, M. K.; Shokat, S.; Inam, S.; Naveed, S. A.; Xu, J.; Li, Z.; Ali, G. M.; Khan, M. R., Impact of pre-anthesis drought stress on physiology, yield-related traits, and drought-responsive genes in green super rice. *Frontiers in Genetics* **2022**, 256.
13. Kojonna, T.; Suttiyut, T.; Khunpolwattana, N.; Pongpanich, M.; Suriya-Arunroj, D.; Comai, L.; Buaboocha, T.; Chadchawan, S., Identification of a negative regulator for salt tolerance at seedling stage via a genome-wide association study of thai rice populations. *International Journal of Molecular Sciences* **2022,** 23, (3), 1842.

14. Huang, L.; Zhang, F.; Zhang, F.; Wang, W.; Zhou, Y.; Fu, B.; Li, Z., Comparative transcriptome sequencing of tolerant rice introgression line and its parents in response to drought stress. *BMC genomics* **2014,** 15, 1-16.
15. Arabia, S.; Sami, A. A.; Akhter, S.; Sarker, R. H.; Islam, T., Comprehensive in silico characterization of universal stress proteins in rice (Oryza sativa L.) with insight into their stress-specific transcriptional modulation. *Frontiers in plant science* **2021,** 12, 712607.
16. Vij, S.; Giri, J.; Dansana, P. K.; Kapoor, S.; Tyagi, A. K., The receptor-like cytoplasmic kinase (OsRLCK) gene family in rice: organization, phylogenetic relationship, and expression during development and stress. *Molecular plant* **2008,** 1, (5), 732-750.
17. Liu, F.; Xu, W.; Song, Q.; Tan, L.; Liu, J.; Zhu, Z.; Fu, Y.; Su, Z.; Sun, C., Microarray-assisted fine-mapping of quantitative trait loci for cold tolerance in rice. *Molecular Plant* **2013,** 6, (3), 757-767.
18. Lv, M.-Z.; Chao, D.-Y.; Shan, J.-X.; Zhu, M.-Z.; Shi, M.; Gao, J.-P.; Lin, H.-X., Rice carotenoid β-ring hydroxylase CYP97A4 is involved in lutein biosynthesis. *Plant and cell physiology* **2012,** 53, (6), 987-1002.
19. Pasion, E. A.; Misra, G.; Kohli, A.; Sreenivasulu, N., Unraveling the genetics underlying micronutrient signatures of diversity panel present in brown rice through genome–ionome linkages. *The Plant Journal* **2023,** 113, (4), 749-771.
20. Wen, P.; He, J.; Zhang, Q.; Qi, H.; Zhang, A.; Liu, D.; Sun, Q.; Wang, Y.; Li, Q.; Wang, W., SET Domain Group 703 Regulates Planthopper Resistance by Suppressing the Expression of Defense-Related Genes. *International Journal of Molecular Sciences* **2023,** 24, (16), 13003.
21. Li, J.; Wang, D.; Sun, S.; Sun, L.; Zong, J.; Lei, Y.; Yu, J.; Liang, W.; Zhang, D., The regulatory role of CARBON STARVED ANTHER-mediated photoperiod-dependent male fertility in rice. *Plant Physiology* **2022,** 189, (2), 955-971.
22. Zheng, Y.; Zhang, S.; Luo, Y.; Li, F.; Tan, J.; Wang, B.; Zhao, Z.; Lin, H.; Zhang, T.; Liu, J., Rice OsUBR7 modulates plant height by regulating histone H2B monoubiquitination and cell proliferation. *Plant Communications* **2022,** 3, (6).
23. Zainal-Abidin, R.-A.; Abu-Bakar, N.; Sew, Y.-S.; Simoh, S.; Mohamed-Hussein, Z.-A., Discovery of Functional SNPs via Genome-Wide Exploration of Malaysian Pigmented Rice Varieties. *International journal of genomics* **2019,** 2019.
24. Xie, Y.; Wang, Y.; Yu, X.; Lin, Y.; Zhu, Y.; Chen, J.; Xie, H.; Zhang, Q.; Wang, L.; Wei, Y., SH3P2, an SH3 domain-containing protein that interacts with both Pib and AvrPib, suppresses effector-triggered, Pib-mediated immunity in rice. *Molecular Plant* **2022,** 15, (12), 1931-1946.
25. Tao, W.; Lijuan, L.; Zeyu, L.; Lianguang, S.; Quan, W., Cloning and characterization of protein prenyltransferase alpha subunit in rice. *Rice Science* **2021,** 28, (6), 557-566.
26. Zheng, K.; Pang, L.; Xue, X.; Gao, P.; Zhao, H.; Wang, Y.; Han, S., Genome-wide comprehensive survey of the subtilisin-like proteases gene family associated with rice caryopsis development. *Frontiers in Plant Science* **2022,** 13, 943184.
27. Collins, N.; Park, R.; Spielmeyer, W.; Ellis, J.; Pryor, A., Resistance gene analogs in barley and their relationship to rust resistance genes. *Genome* **2001,** 44, (3), 375-381.

28. Kim, J.-H.; Yang, D. H.; Kim, J.-S.; Baek, M.-H.; Park, Y. M.; Wi, S. G.; Cho, J.-Y.; Chung, B. Y., Cloning, characterization, and expression of two cDNA clones for a rice ferulate-5-hydroxylase gene, a cytochrome P450-dependent monooxygenase. *Journal of Plant Biology* **2006,** 49, 200-204.
29. Li, J.; Zhang, M.; Sun, J.; Mao, X.; Wang, J.; Wang, J.; Liu, H.; Zheng, H.; Zhen, Z.; Zhao, H., Genome-wide characterization and identification of trihelix transcription factor and expression profiling in response to abiotic stresses in rice (Oryza sativa L.). *International Journal of Molecular Sciences* **2019,** 20, (2), 251.
30. Ueda, M.; Arimura, S.-i.; Yamamoto, M. P.; Takaiwa, F.; Tsutsumi, N.; Kadowaki, K.-i., Promoter shuffling at a nuclear gene for mitochondrial RPL27. Involvement of interchromosome and subsequent intrachromosome recombinations. *Plant physiology* **2006,** 141, (2), 702-710.
31. Yamagata, Y.; Yamamoto, E.; Aya, K.; Win, K. T.; Doi, K.; Sobrizal; Ito, T.; Kanamori, H.; Wu, J.; Matsumoto, T., Mitochondrial gene in the nuclear genome induces reproductive barrier in rice. *Proceedings of the National Academy of Sciences* **2010,** 107, (4), 1494-1499.
32. Li, W.; Yoshida, A.; Takahashi, M.; Maekawa, M.; Kojima, M.; Sakakibara, H.; Kyozuka, J., SAD1, an RNA polymerase I subunit A34. 5 of rice, interacts with Mediator and controls various aspects of plant development. *The Plant Journal* **2015,** 81, (2), 282-291.
33. Fatihi, A.; Latimer, S.; Schmollinger, S.; Block, A.; Dussault, P. H.; Vermaas, W. F.; Merchant, S. S.; Basset, G. J., A Dedicated Type II NADPH Dehydrogenase Performs the Penultimate Step in the Biosynthesis of Vitamin K1 in S ynechocystis and Arabidopsis. *The Plant Cell* **2015,** 27, (6), 1730-1741.
34. Carrie, C.; Murcha, M. W.; Kuehn, K.; Duncan, O.; Barthet, M.; Smith, P. M.; Eubel, H.; Meyer, E.; Day, D. A.; Millar, A. H., Type II NAD (P) H dehydrogenases are targeted to mitochondria and chloroplasts or peroxisomes in Arabidopsis thaliana. *FEBS letters* **2008,** 582, (20), 3073-3079.
35. Singh, A.; Kumar, P.; Gautam, V.; Rengasamy, B.; Adhikari, B.; Udayakumar, M.; Sarkar, A. K., Root transcriptome of two contrasting indica rice cultivars uncovers regulators of root development and physiological responses. *Scientific Reports* **2016,** 6, (1), 39266.
36. Chen, D.; Qiu, Z.; He, L.; Hou, L.; Li, M.; Zhang, G.; Wang, X.; Chen, G.; Hu, J.; Gao, Z., The rice LRR-like1 protein YELLOW AND PREMATURE DWARF 1 is involved in leaf senescence induced by high light. *Journal of Experimental Botany* **2021,** 72, (5), 1589-1605.
37. Yang, X.; Xia, X.; Zeng, Y.; Nong, B.; Zhang, Z.; Wu, Y.; Tian, Q.; Zeng, W.; Gao, J.; Zhou, W., Genome-wide identification of the peptide transporter family in rice and analysis of the PTR expression modulation in two near-isogenic lines with different nitrogen use efficiency. *BMC plant biology* **2020,** 20, 1-15.
38. Syarifudin, A.; Chadchawan, S., Potential Role of Peptide Transporter2 Gene in Salt Stress. *Genomics and Genetics* **2022,** 15, (2), 55-63.
39. Singh, A.; Giri, J.; Kapoor, S.; Tyagi, A. K.; Pandey, G. K., Protein phosphatase complement in rice: genome-wide identification and transcriptional analysis under abiotic stress conditions and reproductive development. *BMC genomics* **2010,** 11, (1), 1-18.

40. Zhang, A.; Zhang, W., Characterization of Transposon-Derived Accessible Chromatin Regions in Rice (Oryza sativa). *International Journal of Molecular Sciences* **2022,** 23, (16), 8947.
41. Hu, X.; Chen, G.; Zhang, R.; Xu, M.; Zhao, L.; Tang, H.; Ni, J.; Zhou, M., Multi-Year QTL Mapping and RNA-seq Reveal Candidate Genes for Early Floret-Opening Time in Japonica Rice. *Agriculture* **2023,** 13, (4), 859.
42. Ke, S.; Liu, X.-J.; Luan, X.; Yang, W.; Zhu, H.; Liu, G.; Zhang, G.; Wang, S., Genome-wide transcriptome profiling provides insights into panicle development of rice (Oryza sativa L.). *Gene* **2018,** 675, 285-300.
43. Sinha, S. K.; V, A. M. S.; Chaudhary, S.; Tyagi, P.; Venkadesan, S.; Rani, M.; Mandal, P. K., Transcriptome analysis of two rice varieties contrasting for nitrogen use efficiency under chronic N starvation reveals differences in chloroplast and starch metabolism-related genes. *Genes* **2018,** 9, (4), 206.
44. Ontoy, J. C. E., *Genetic Characterization of Resistance to Bacterial Panicle Blight and Sheath Blight in Rice Using QTL Linkage Analysis and QTL-SEQ*. Louisiana State University and Agricultural & Mechanical College: 2022.
45. Manzanares, C. Genetics of self-incompatibility in perennial ryegrass (Lolium perenne L.). University of Birmingham, 2013.
46. Ma, Y.; Mackon, E.; Jeazet Dongho Epse Mackon, G. C.; Zhao, Y.; Li, Q.; Dai, X.; Yao, Y.; Xia, X.; Nong, B.; Liu, P., Combined Analysis of BSA-Seq Based Mapping, RNA-Seq, and Metabolomic Unraveled Candidate Genes Associated with Panicle Grain Number in Rice (Oryza sativa L.). *Biomolecules* **2022,** 12, (7), 918.
47. Bakade, R.; Ingole, K. D.; Deshpande, S.; Pal, G.; Patil, S. S.; Bhattacharjee, S.; Prasannakumar, M.; Ramu, V. S., Comparative transcriptome analysis of rice resistant and susceptible genotypes to Xanthomonas oryzae pv. oryzae identifies novel genes to control bacterial leaf blight. *Molecular Biotechnology* **2021,** 63, (8), 719-731.
48. Dauda, W. P.; Shanmugam, V.; Tyagi, A.; Solanke, A. U.; Kumar, V.; Krishnan, S. G.; Bashyal, B. M.; Aggarwal, R., Genome-wide identification and characterisation of cytokinin-O-glucosyltransferase (CGT) genes of rice specific to potential pathogens. *Plants* **2022,** 11, (7), 917.
49. Duan, W.; Xue, B.; He, Y.; Liao, S.; Li, X.; Li, X.; Liang, Y.-K., Genome-Wide Identification and Expression Pattern Analysis of Dirigent Members in the Genus Oryza. *International Journal of Molecular Sciences* **2023,** 24, (8), 7189.
50. Guo, X.; Fu, Y.; Lee, Y. R. J.; Chern, M.; Li, M.; Cheng, M.; Dong, H.; Yuan, Z.; Gui, L.; Yin, J., The PGS1 basic helix-loop-helix protein regulates Fl3 to impact seed growth and grain yield in cereals. *Plant biotechnology journal* **2022,** 20, (7), 1311-1326.
51. Vejchasarn, P., *Nutritional and genetic architecture of root traits in rice (Oryza sativa)*. The Pennsylvania State University: 2014.
52. Ouyang, Y.; Huang, X.; Lu, Z.; Yao, J., Genomic survey, expression profile and co-expression network analysis of OsWD40 family in rice. *BMC genomics* **2012,** 13, 1-15.
53. Wang, J.; Wang, J.; Wang, X.; Li, R.; Chen, B., Proteomic response of hybrid wild rice to cold stress at the seedling stage. *PLoS One* **2018,** 13, (6), e0198675.

54. Hayama, R.; Izawa, T.; Shimamoto, K., Isolation of rice genes possibly involved in the photoperiodic control of flowering by a fluorescent differential display method. *Plant and cell physiology* **2002,** 43, (5), 494-504.
55. Hayama, R.; Yokoi, S.; Tamaki, S.; Yano, M.; Shimamoto, K., Adaptation of photoperiodic control pathways produces short-day flowering in rice. *Nature* **2003,** 422, (6933), 719-722.
56. Li, S.; Yue, W.; Wang, M.; Qiu, W.; Zhou, L.; Shou, H., Mutation of OsGIGANTEA leads to enhanced tolerance to polyethylene glycol-generated osmotic stress in rice. *Frontiers in Plant Science* **2016,** 7, 465.
57. Goh, C.-H.; Jung, K.-H.; Roberts, S. K.; McAinsh, M. R.; Hetherington, A. M.; Park, Y.-i.; Suh, K.; An, G.; Nam, H. G., Mitochondria provide the main source of cytosolic ATP for activation of outward-rectifying K+ channels in mesophyll protoplast of chlorophyll-deficient mutant rice (OsCHLH) seedlings. *Journal of Biological Chemistry* **2004,** 279, (8), 6874-6882.
58. Sheng, Z.; Lv, Y.; Li, W.; Luo, R.; Wei, X.; Xie, L.; Jiao, G.; Shao, G.; Wang, J.; Tang, S., Yellow-Leaf 1 encodes a magnesium-protoporphyrin IX monomethyl ester cyclase, involved in chlorophyll biosynthesis in rice (Oryza sativa L.). *PLoS One* **2017,** 12, (5), e0177989.
59. Higuchi-Takeuchi, M.; Ichikawa, T.; Kondou, Y.; Matsui, K.; Hasegawa, Y.; Kawashima, M.; Sonoike, K.; Mori, M.; Hirochika, H.; Matsui, M., Functional analysis of two isoforms of leaf-type ferredoxin-NADP+-oxidoreductase in rice using the heterologous expression system of Arabidopsis. *Plant physiology* **2011,** 157, (1), 96-108.
60. Piao, W.; Kim, E.-Y.; Han, S.-H.; Sakuraba, Y.; Paek, N.-C., Rice phytochrome B (OsPhyB) negatively regulates dark-and starvation-induced leaf senescence. *Plants* **2015,** 4, (3), 644-663.
61. Lin, A.; Wang, Y.; Tang, J.; Xue, P.; Li, C.; Liu, L.; Hu, B.; Yang, F.; Loake, G. J.; Chu, C., Nitric oxide and protein S-nitrosylation are integral to hydrogen peroxide-induced leaf cell death in rice. *Plant Physiology* **2012,** 158, (1), 451-464.
62. Zhao, Q.; Shi, X.-S.; Wang, T.; Chen, Y.; Yang, R.; Mi, J.; Zhang, Y.-W.; Zhang, Y.-M., Identification of QTNs, QTN-by-environment interactions, and their candidate genes for grain size traits in main crop and ratoon rice. *Frontiers in Plant Science* **2023,** 14, 1119218.
63. Wu, Q.; Han, T.; Yang, L.; Wang, Q.; Zhao, Y.; Jiang, D.; Ruan, X., The essential roles of OsFtsH2 in developing the chloroplast of rice. *BMC Plant Biology* **2021,** 21, (1), 1-14.
64. Diédhiou, C.; Popova, O.; Dietz, K. J.; Golldack, D., The SUI-homologous translation initiation factor eIF-1 is involved in regulation of ion homeostasis in rice. *Plant Biology* **2008,** 10, (3), 298-309.
65. Rangan, L.; Rout, A.; Sudarshan, M.; Gregorio, G., Molecular cloning, expression and mapping of the translational initiation factor eIF1 gene in Oryza sativa. *Functional Plant Biology* **2009,** 36, (5), 442-452.
66. Hu, W.; Zhou, T.; Hu, G.; Wu, H.; Han, Z.; Xiao, J.; Li, X.; Xing, Y., An ethyl methanesulfonate-induced neutral mutant-bridging method efficiently identifies spontaneously mutated genes in rice. *The Plant Journal* **2020,** 104, (4), 1129-1141.
67. Mekawy, A. M. M.; Assaha, D. V.; Ueda, A., Constitutive overexpression of rice metallothionein-like gene OsMT-3a enhances growth and tolerance of Arabidopsis

plants to a combination of various abiotic stresses. *Journal of plant research* **2020,** 133, 429-440.

68. Yadavalli, V.; Neelam, S.; Rao, A. S.; Reddy, A. R.; Subramanyam, R., Differential degradation of photosystem I subunits under iron deficiency in rice. *Journal of plant physiology* **2012,** 169, (8), 753-759.
69. Qiu, Z.; Chen, D.; Teng, L.; Guan, P.; Yu, G.; Zhang, P.; Song, J.; Zeng, Q.; Zhu, L., OsWHY1 Interacts with OsTRX z and is Essential for Early Chloroplast Development in Rice. *Rice* **2022,** 15, (1), 50.
70. Song, A.; Li, P.; Fan, F.; Li, Z.; Liang, Y., The effect of silicon on photosynthesis and expression of its relevant genes in rice (Oryza sativa L.) under high-zinc stress. *PLoS One* **2014,** 9, (11), e113782.
71. Chen, X.; Yu, T.; Xiong, J.; Zhang, Y.; Hua, Y.; Li, Y.; Zhu, Y., Molecular cloning and expression analysis of rice phosphoribulokinase gene that is regulated by environmental stresses. *Molecular biology reports* **2005,** 31, 249-255.
72. Chen, T.; Wu, H.; Wu, J.; Fan, X.; Li, X.; Lin, Y., Absence of OsβCA1 causes a CO2 deficit and affects leaf photosynthesis and the stomatal response to CO2 in rice. *The Plant Journal* **2017,** 90, (2), 344-357.
73. Chintakovid, N.; Maipoka, M.; Phaonakrop, N.; Mickelbart, M. V.; Roytrakul, S.; Chadchawan, S., Proteomic analysis of drought-responsive proteins in rice reveals photosynthesis-related adaptations to drought stress. *Acta Physiologiae Plantarum* **2017,** 39, 1-13.
74. Wang, D.; Xu, T.; Yin, Z.; Wu, W.; Geng, H.; Li, L.; Yang, M.; Cai, H.; Lian, X., Overexpression of OsMYB305 in rice enhances the nitrogen uptake under low-nitrogen condition. *Frontiers in plant science* **2020,** 11, 369.
75. Li, X.; Duan, X.; Jiang, H.; Sun, Y.; Tang, Y.; Yuan, Z.; Guo, J.; Liang, W.; Chen, L.; Yin, J., Genome-wide analysis of basic/helix-loop-helix transcription factor family in rice and Arabidopsis. *Plant physiology* **2006,** 141, (4), 1167-1184.
76. Yang, X.; Ren, Y.; Cai, Y.; Niu, M.; Feng, Z.; Jing, R.; Mou, C.; Liu, X.; Xiao, L.; Zhang, X., Overexpression of OsbHLH107, a member of the basic helix-loop-helix transcription factor family, enhances grain size in rice (Oryza sativa L.). *Rice* **2018,** 11, (1), 1-12.
77. Xie, Y.; Niu, B.; Long, Y.; Li, G.; Tang, J.; Zhang, Y.; Ren, D.; Liu, Y. G.; Chen, L., Suppression or knockout of SaF/SaM overcomes the Sa-mediated hybrid male sterility in rice. *Journal of integrative plant biology* **2017,** 59, (9), 669-679.
78. Long, Y.; Zhao, L.; Niu, B.; Su, J.; Wu, H.; Chen, Y.; Zhang, Q.; Guo, J.; Zhuang, C.; Mei, M., Hybrid male sterility in rice controlled by interaction between divergent alleles of two adjacent genes. *Proceedings of the National Academy of Sciences* **2008,** 105, (48), 18871-18876.
79. Jain, M.; Nijhawan, A.; Arora, R.; Agarwal, P.; Ray, S.; Sharma, P.; Kapoor, S.; Tyagi, A. K.; Khurana, J. P., F-box proteins in rice. Genome-wide analysis, classification, temporal and spatial gene expression during panicle and seed development, and regulation by light and abiotic stress. *Plant physiology* **2007,** 143, (4), 1467-1483.

80. Li, H.; Liang, Z.; Ding, G.; Shi, L.; Xu, F.; Cai, H., A natural light/dark cycle regulation of carbon-nitrogen metabolism and gene expression in rice shoots. *Frontiers in Plant Science* **2016,** 7, 1318.
81. Huang, S.; Xin, S.; Xie, G.; Han, J.; Liu, Z.; Wang, B.; Zhang, S.; Wu, Q.; Cheng, X., Mutagenesis reveals that the rice OsMPT3 gene is an important osmotic regulatory factor. *The Crop Journal* **2020,** 8, (3), 465-479.
82. Park, Y. C.; Kim, J. J.; Kim, D. S.; Jang, C. S., Rice RING E3 ligase may negatively regulate gamma-ray response to mediate the degradation of photosynthesis-related proteins. *Planta* **2015,** 241, 1119-1129.
83. Patishtan Perez, J. Genome Wide Association and Forward Genetic Studies to Identify Genes Involved in Salt Tolerance. University of York, 2016.
